# Synergistic Effect of Ternary Substitution in Na_3_V_2_(PO_4_)_3_ for High-Rate and Long-Life Anode-Free SIBs

**DOI:** 10.1021/acsami.5c08411

**Published:** 2025-07-21

**Authors:** Ruoyu Chen, Dongdong Li, Xinyu Zhang, Shuoxiao Zhang, Xingyu Wang, Shilin Li, Il’ya A. Gural’skiy, Igor V. Zatovsky, Wei Han, Denys Butenko

**Affiliations:** † School of Mechanical and Intelligent Manufacturing, 519822 Fujian Chuanzheng Communications College, Fuzhou 350007, China; ‡ College of Physics, The State Key Laboratory of Inorganic Synthesis and Preparative Chemistry, International Center of Future Science, 12510 Jilin University, Changchun 130012, China; § Department of Physics and Institute of Major Scientific Facilities for New Materials, 255310 Southern University of Science and Technology, Shenzhen 518055, China; ∥ Department of Physics, 53025 City University of Hong Kong, Kowloon, Hong Kong 999077, China; ⊥ Department of Chemistry, Taras Shevchenko National University of Kyiv, Kyiv 01601, Ukraine; # F.D. Ovcharenko Institute of Biocolloidal Chemistry, NAS Ukraine, Kyiv 03142, Ukraine

**Keywords:** SIB, cathode materials, NASICON-type, NVP, sol−gel synthesis

## Abstract

Doping with metal ions can significantly enhance the electrochemical performance of Na_3_V_2_(PO_4_)_3_ (NVP) as a cathode material for sodium-ion batteries (SIBs). Despite its high reversible capacity and high voltage, practical application of NVP is limited by its poor intrinsic conductivity. Herein, we shed light on a facile sol–gel synthesis method to prepare NVP with ternary doping of potassium K^+^, Al^3+^, and SO_4_^2–^ ions, which accelerates the migration of Na^+^ in the crystal structure, as confirmed by theoretical calculations. The new cathode materials exhibit a notable reversible capacity of 115.3 mA·h g^–1^ at 0.5C and retain a substantial initial capacity of 79.1 mA·h g^–1^ even at 50C. Additionally, we synthesized NVP with the doping of SO_4_^2–^ ion on the PO_4_^3–^ site, which shows 90.6 and 85 mA·h g^–1^ at 0.5 and 10C, respectively. These results perfectly demonstrate the advantages of the superiority of ternary substitution on the NVP cathode. Moreover, sodium anode-free full cells of the cathode paired with Cu/SP foil showed impressive long-term cycling stability. This achievement lays a strong foundation for the use of NASICON cathodes with high charge–discharge rates and high energy density, making them a promising alternative to the next-generation lithium-ion batteries.

## Graphical abstract

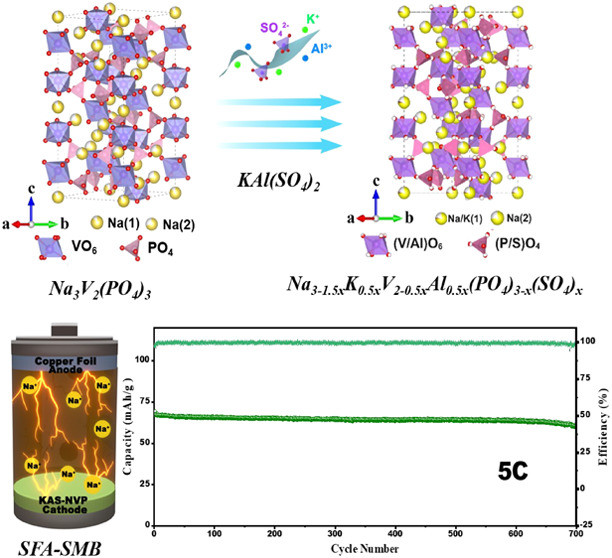

## Introduction

Energy is an important basis for social progress and technological development. With today’s increasingly severe environmental pollution and energy shortages, the development and large-scale use of renewable clean energy has become one of the most important research topics. Achieving carbon neutrality has become a priority goal for many countries, highlighting the need for efficient and environmentally friendly energy storage solutions.^1–3^ Lithium-ion batteries (LIB) stand out among numerous energy storage devices due to their high energy density, long service life, and lack of a memory effect. However, the limited and uneven geographical distribution of lithium resources creates new challenges.^4–6^ In this context, sodium-ion batteries (SIBs) represent a promising alternative since sodium is more available and evenly distributed.^7–9^ However, sodium-ion batteries face obstacles, such as lower energy density, electrochemical stability, and electrode dynamics, which are directly dependent on the properties of the cathode materials. In addition, an important aspect of the widespread use of sodium-ion batteries is the ability to quickly charge, which can significantly increase their competitiveness and practical value.^10^

At present, the SIB cathode materials studied mainly include transition metal oxides, polyanionic compounds, Prussian blue derivatives, and organic cathode materials. Through comparative research, the natrium super ionic conductor (NASICON) type polyanion compounds have attracted great interest due to their stable three-dimensional (3D) open phosphate skeleton structure, rich crystal structure diversity, and high working potential.^11–14^ Na_3_V_2_(PO4)_3_ (NVP), as the most basic NASICON-type cathode, has been widely studied. NVP not only has a stable operating voltage platform (3.4 V), high energy density (400 Wh kg^–1^), and high theoretical capacity (117.6 mAh g^–1^), but it also dominates the controllable NASICON-typed system, establishing favorable channels for transition metal ion conversion and sodium-ion de/intercalation.^15–18^ In recent years, many studies have been devoted to improving the electrochemical performance of NVP or similar NASICON phosphates, such as special nanostructure design,^16,19–21^ composite with highly conductive carbon materials,^22–24^ or the introduction of other heteroions (heteroion doping)^25–30^ in the NASICON-typed structure. Through a comparative study, the doping of the NASICON-typed structure is a simple and effective method to improve performance. The NVP doping type can be divided into Na-site,^25–27^ V-site,^28–33^ and PO_4_^3–^-site doping^34,35^ according to the different doping sites of NVP, and also can be distinguished as single-ion-doped^25,26,28–36^ and multi-ions doped^27,37–39^ according to the types of doping ions. So far, the research on microdoped NVP has mainly focused on V-site single-ion doping, and there are few reports on multi-ion doping.

The V-site and Na-site of NVP are generally doped with metal cations, such as Li^+^,^25^ K^+^,^26^ Mg^2+^,^28^ Al^3+^,^29^ Mn^2+^,^30^ Ni^2+^,^31^ Fe^3+^,^32^ Cu^2+^,^33^ Co^2+^,^33^ Sn^4+^,^40,41^ and so on. On the one hand, it helps to improve the electronic conductivity of the material. On the other hand, it can improve the lattice structure of NVP, thereby enhancing the transmission of Na^+^ in the material. Doping Sn^4+^ ions into the Na_3_V_2_(PO_4_)_3_ lattice improves the intrinsic electronic conductivity, enabling a stable capacity of 76 mAh g^–1^ after 3000 cycles at 20C.^41^ Codoping with K^+^ and Ca^2+^ expands the unit cell volume and enhances the structural stability of the NASICON framework, delivering reversible capacities of 110.2, 92.7, and 83.6 mAh g^–1^ at current densities of 0.1, 1, and 10C, respectively.^27^ For the PO_4_^3–^ site doping of NVP, the doped ions generally use F^–^,^34^ SiO_4_^4–^ and other anions. Chen et al. successfully synthesized a NASICON-type F-ion-doped Na_3_V_2_(PO_4_)_2.93_F_0.07_/C cathode via a high-temperature solid-phase method. The doping of F can effectively suppress the structural degradation caused by the adverse phase transition of NVP during charge and discharge processes and increase its structural stability. The optimized positive electrode can provide a reversible capacity of 113 at 10 mA g^–1^, which is very close to the theoretical capacity of NVP (117 mAh g^–1^), and can still maintain 86% capacity after 1000 cycles at 200 mA g^–1^.^34^ It should be noted that multicomponent doping is one of the most promising ways to improve the desired characteristics of various materials for electrochemical applications.^42^ In the case of NVP, although single-ion micro doping can effectively improve the electrochemical capacity and cycling performance of NVP at low currents by improving its conductivity and lattice structure, the improvement in high-rate characteristics and long-term cycling life is not particularly significant. Therefore, the synergistic effect generated by multi-ion doping of NVP can be considered to further enhance the stability of the material crystal structure and electronic conductivity, effectively solve the slow sodium storage kinetics of the electrode, and improve the electrochemical performance of the material.^43,44^ In particular, according to Shiqi Sun et al.’s report, a series of Na_3.1–*x*_K_*x*_V_2–*x*_La_*x*_(PO_4_)_2.9_(SiO_4_)_0.1_ (KLS*x*, *x* = 0.01–0.1) cathode materials were successfully prepared by doping K^+^, La^3+,^ and SiO_4_^4–^ by the sol–gel method.^39^ Under the combined action of ternary ion doping, the crystal undergoes a slight lattice distortion, which not only enhances conductivity but also releases more active sites for the reversible deintercalation/intercalation of Na^+^. Therefore, the KLS0.07 sample has a high reversible capacity of 115.7 mAh g^–1^, and its initial capacity can reach 84.8 mAh g^–1^ at a high rate of 50C. After 1300 cycles, it still maintains 69.8% of the capacity.

In this work, we used the double sulfate KAl­(SO_4_)_2_·12H_2_O for a simple and effective method of multiionic and controlled simultaneous doping of different sites of the NVP matrix. A series of cathodes (KAS_*X*-NVP) with the general composition Na_3–1.5*x*_K_0.5*x*_V_2–0.5*x*_Al_0.5*x*_(PO_4_)_3–*x*_(SO_4_)_*x*_ (*x* = 0–0.06) were successfully prepared by the sol–gel method. Generally, multi-ion doping necessitates the use of multiple additives during the synthesis process. In this study, the simultaneous doping and regulation of Na, V, and PO_4_^3–^ sites in NVP was achieved using the double sulfate KAl­(SO_4_)_2_·12H_2_O. Trace Al^3+^ doping effectively enhanced the electronic conductivity of NVP, thereby improving its cycling performance. As K^+^ ions exhibit minimal involvement in electrochemical extraction-insertion reactions, they mitigate significant lattice volume changes and structural deformations in NVP, enhancing structural stability. Although SO_4_^2–^ ions, as anionic species, do not significantly contribute to improving the electronic conductivity of the NVP cathode, their incorporation into the NASICON-type phosphate framework further enhances the rate capability and cycle life of NVP. The synergistic effect generated by multi-ions doping of NVP can be considered to enhance further the crystal structure stability and electronic conductivity of the material, effectively solving the slow sodium storage kinetics and electrochemical performance of NASICON-typed compounds. Benefiting from the synergistic effect of ternary ion codoping, the electrochemical performance of the prepared KAS_4-NVP cathode is significantly superior to the NASICON-type positive electrode reported in the literature. the KAS_4-NVP cathode with an initial capacity of 115.3 mAh g^–1^ at 0.5C (close to the theoretical capacity of NVP) has the best characteristics. Moreover, there are still 90 and 72.8% high-capacity retention rates after 1000 and 4000 cycles at 10 and 50C. GITT, CV, and EIS tests show that the KAS_4-NVP cathode has an excellent ability for fast sodium-ion transport and electrode kinetics. In addition, sodium-free-anode sodium metal batteries (SFA-SMBs) assembled with KAS_4-NVP cathode and Cu/SP foil also exhibit excellent electrochemical performance. Here, efforts are made to explore the synergistic effect of multi-ion doping on NVP to contribute to the development and application of NASICON-type cathode with a high charge–discharge rate coupled with high energy density, making it a viable next-generation alternative to Li-ion batteries.

## Results and Discussion

The Sketch Map of the synthesis for the KAS-NVP samples is shown in Figure 1. Different sites of NVP were doped with cheap (KAl­(SO_4_)_2_·12H_2_O) by the sol–gel method. Based on thermogravimetry analysis presented in previous work by Wang W.,^45^ the precursor is preheated at 350 °C, and each component forms a solid solution through solid-state reaction. During the preheating stage at 350 °C, the precursors decompose in a controlled manner, avoiding rapid gas release at higher temperatures, which might otherwise lead to structural collapse or morphology disruption. Then, it is calcined at 750 °C for 8 h to form a NASICON-type structure. The VO_6_ octahedra and PO_4_ tetrahedra rearrange via solid-state reaction to form the NASICON-type crystal framework. At the same time, citric acid carbonization forms an amorphous carbon coating on the surface of the material to form a carbon layer, increasing the conductivity of the material. The modification of the NVP lattice structure by Al^3+^, K^+^, and SO_4_^2–^ ions was helpful in improving the lattice stability and electrochemical performance of NVP materials. In general, the crystal structure of NVP contains three types of available cationic sites for doping with different elements. These include the V^–^ (cation) and P-sites (PO^4–^ anions) of the open three-dimensional anionic framework [V_2_(PO_4_)_3_]^3–^ and the Na1 site (Wyckoff position 6b). As for the Na2 site (Wyckoff position 18e), its doping is not characteristic (except for Li^+^), but the filling or occurrence of a vacancy in the Na2 sites significantly expands the possibilities of multivalent doping for the other three crystallographic positions.

**Figure 1:**
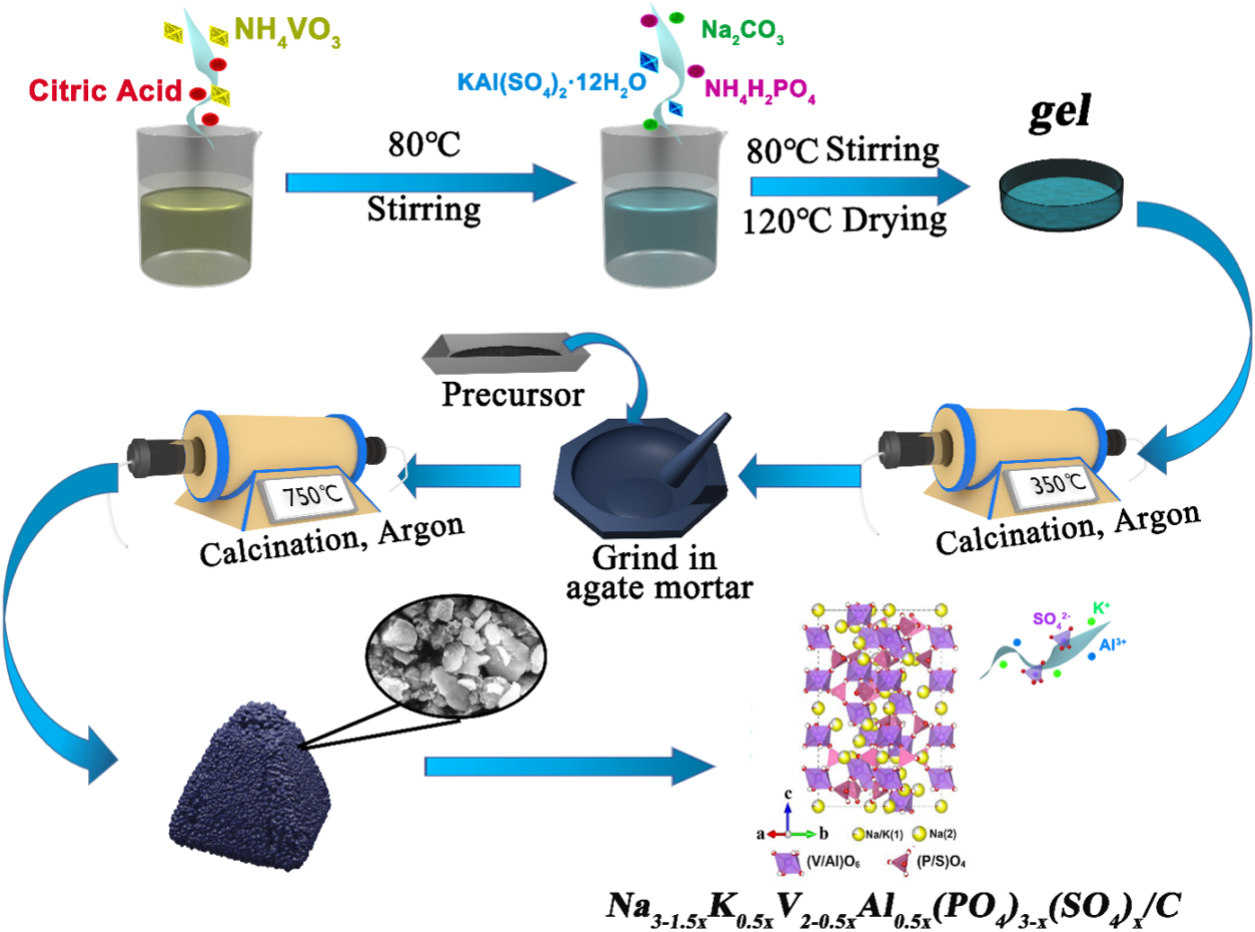
Sketch map of the preparation of Na_3–1.5*x*_K_0.5*x*_V_2–0.5*x*_Al_0.5*x*_(PO_4_)_3–*x*_(SO_4_)_*x*_/C by the sol–gel method.

Based on these principles, we considered a simple approach for multiple-doping the NVP matrix using double sulfate KAl­(SO_4_)_2_·12H_2_O, which involved controlled doping of K to the Na1 site, Al to the V-site, and S to the P-site. (Figure 2a). For comparison, a series of samples with only P-site doping with sulfur was also prepared. The synthesis was carried out using the sol–gel method in accordance with eqs 1 and 2 (Experimental Section). According to the XRD results, the prepared samples are structural analogues of NVP, and all the observed diffraction peaks can be indexed to the *R*3̅*c* space group (Figure 2b). Rietveld refinement of the structures was performed from XRD patterns using the crystal structure of Na_3_V*_2_* (PO_4_)_3_ as a starting structural model.^46^ It should be emphasized that the geometries of PO_4_ and SO_4_ tetrahedra are different due to local changes in the oxygen environment of phosphorus and sulfur atoms. Therefore, we first modified the original structural model by highlighting additional crystallographic O1b and O2b sites related to the sulfur content of the sample (detailed in SI). The final results of the Rietveld refinement are shown in Figures 2c andS1, which are listed in Tables S1–S7. For all samples, the principles of building a crystal framework are similar to NVP; the structures can be described as an anionic framework [(V/Al)_2_(P/S)_3_O_12_]^*x*−^, which forms 3D interconnected channels with two types of internodes positions, namely a shared Na1/K1/vacancy position and a Na2/vacancy position (Figure 2d).

**Figure 2:**
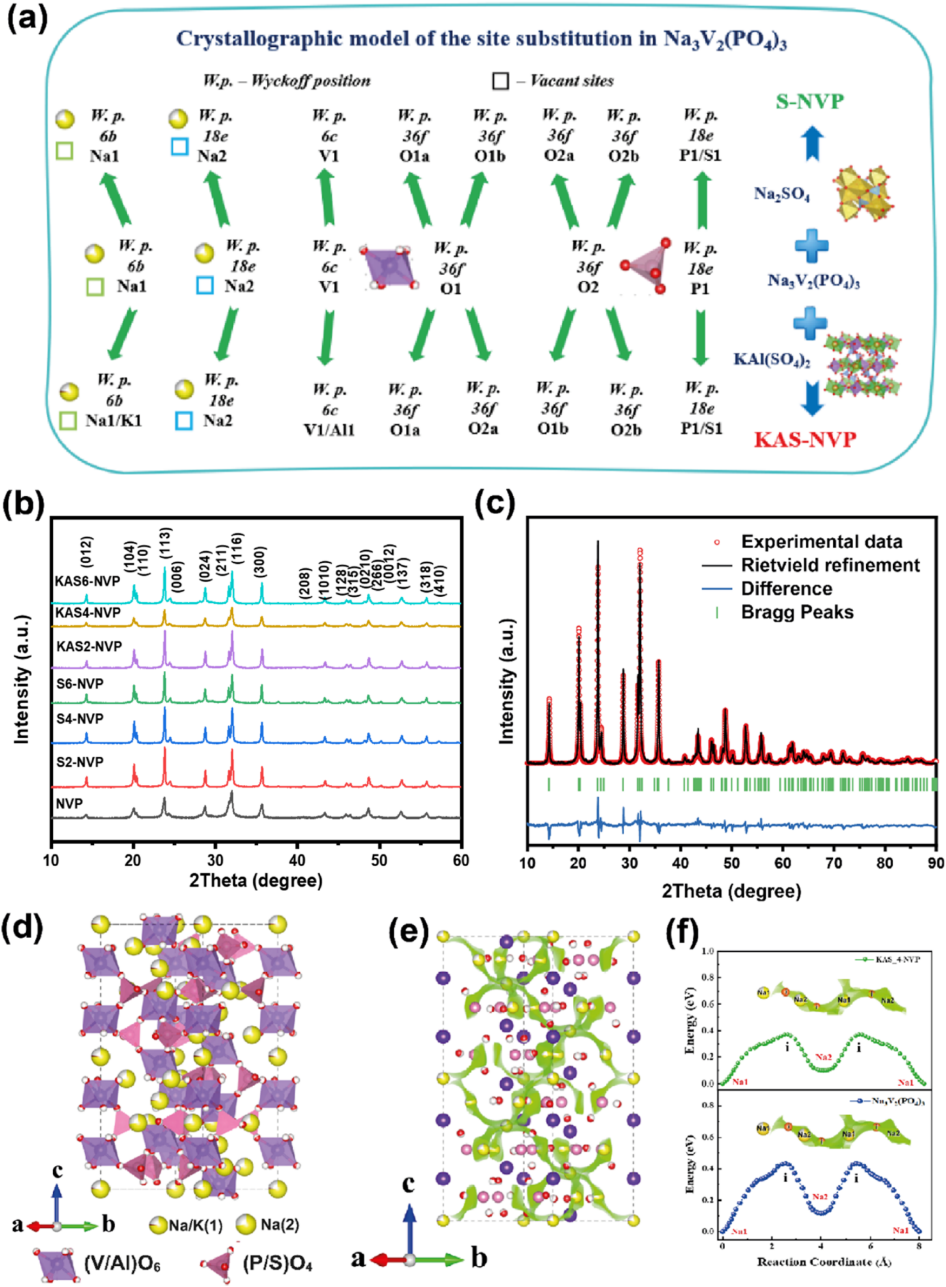
(a) Schematic representation of site substitution in NVP, (b) PXRD patterns of unsubstituted NVP and two series of substituted NVP samples, (c) rietveld refined XRD patterns for KAS_4-NVP, (d) general representation of a substituted NVP structure showing Na/K, V/Al and P/S site sharing, (e) BVSE maps showing 3D migration pathways for KAS_4-NVP, and (f) BVSE models for migration energy barriers for KAS_4-NVP and NVP.

It should be noted that K ions are not removed from the framework during the desodiation process. Thus, only sodium ions will be mobile. Therefore, the Na-ion transport pathways in crystalline ion conductors were analyzed by the bond valence site energy (BVSE) method.^47,48^ The Na-ion pathway shows a three-dimensional network of pathways that is based on the hopping of Na^+^ ions between Na(1) and Na(2).^37,49,50^ As a rule, the Na1 position is more energetically favorable, having a more stable coordination environment and lower local stresses. In the case of the Na2 position, the site is characterized by a higher energy due to a less stable coordination environment or higher local stresses. Na ions at this position have a higher potential energy, making them less stable and more prone to migration. As the calculation shows, the most probable migration paths lie along the *c* axis between Na1–Na2–Na1 sites with diffusion barriers of 0.434 and 0.369 eV for NVP and KAS_4-NVP samples, respectively (Figure 2e,f). It is interesting that in all cases, there are additional saddle points (i) along the migration path of Na ions. The presence of saddle points (i) leads to a local disturbance of the node energy and creates a distribution of node energy, which leads to energy overlap between adjacent migration sites and facilitates the transition of ions between them. According to the calculation results, the saddle point is located at a distance of 2.5534A from the Na1 position in the case of the NVP sample, and for the KAS_4-NVP sample it is located at a distance of 2.6457A, which is closer to the Na2 position. The proximity of saddle points to the Na2 position in the NASICON structure leads to a decrease in energy barriers, and an increase in the probability of transitions of Na ions directly leads to an increase in the ionic conductivity of the cathode materials. This makes KAS_4-NVP a more efficient conductor for the migration of sodium ions, which is directly proportional to its high electrochemical performance.

The SEM images of KAS-NVP series samples are shown in Figures 3a andS2. The microstructure of pure NVP is very similar to that of all ternary ion-doped KAS-NVP samples. They are mainly composed of irregular block-like particles. However, the particle size of the doped samples is about 1–3 μm, which is significantly smaller than pure NVP. The smaller material particle size accelerates electron transfer between particles during the charging–discharging process. Meanwhile, obvious K, Al, and S signals can be observed in the EDS element mapping results of the KAS_4-NVP sample (Figure 3a), which confirms the successful doping of K^+^, Al^3+^, and SO_4_^2–^ ternary ions. All elements of the KAS_4-NVP sample are evenly distributed in the region, indicating that the as-prepared sample has high homogeneity. A similar microstructure and uniform distribution of elements are also observed for samples of the S-NVP series (Figure S3). The clear lattice stripes can be observed in the High resolution-TEM (HR-TEM) images of the KAS_4-NVP sample (Figure 3b,c), and the *d*-spacings of the crystal bands with values of 0.374, 0.285, and 0.443 nm are consistent with the band spacings of the crystal lattice (113), (211) and (104) planes, respectively. In addition, due to the incomplete combustion of citric acid in a high-temperature inert atmosphere, the surface of KAS_4-NVP material is uniformly covered by an amorphous carbon coating with a thickness of 3–6 nm, which helps to increase the electronic conductivity,^51^ thus significantly improving the electrochemical performance of the electrode.

**Figure 3:**
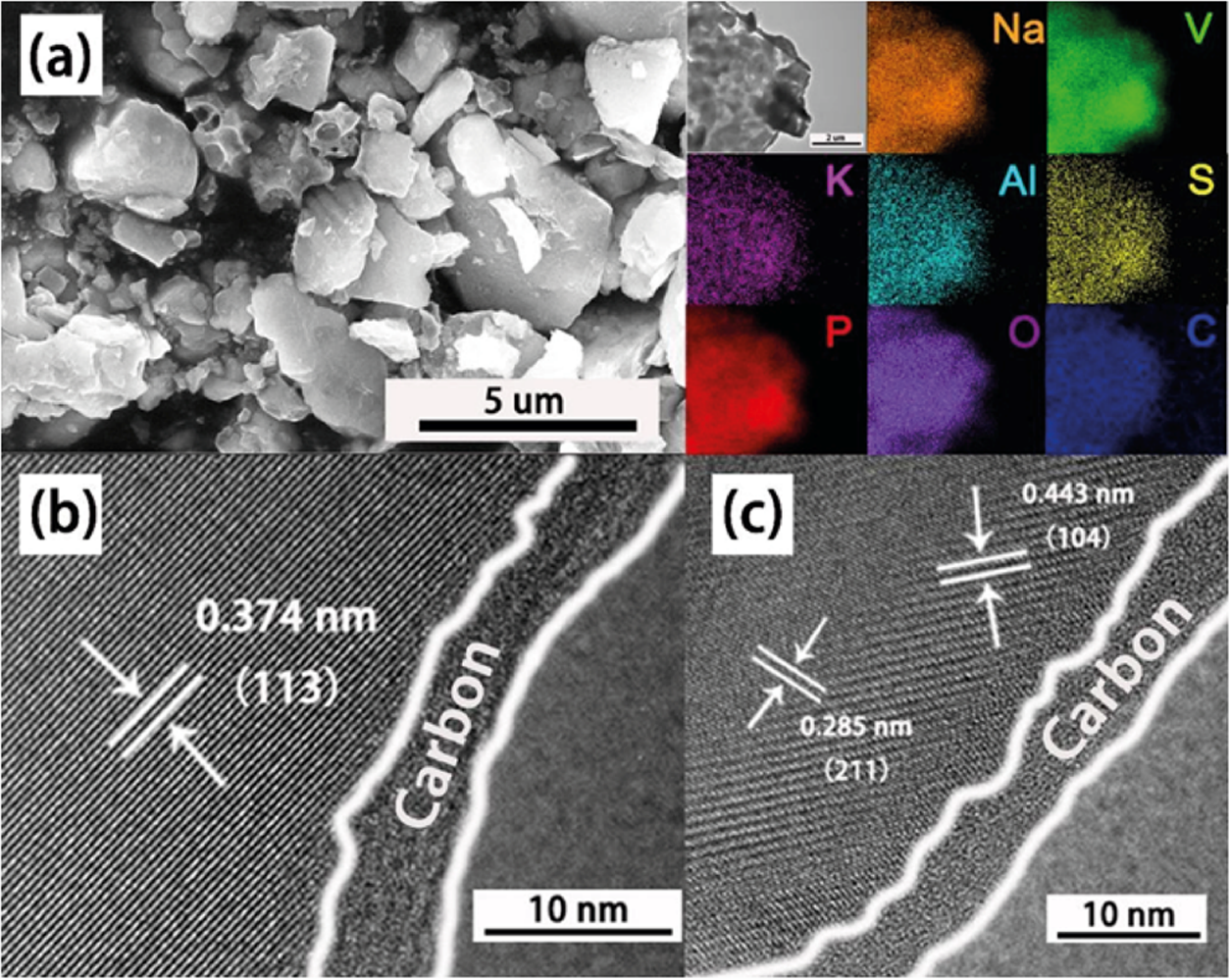
Microstructure of KAS_4-NVP sample: (a) SEM image and EDS element mapping and (b, c) HR-TEM images.

The surface carbon layer structure of the material particles is further analyzed by the Raman test (Figure 4a). In the Raman spectra of KAS_4-NVP and pure NVP samples, it is clear that there are three distinct wide peaks at 1355, 1586, and 2907 cm^–1^, which correspond to the D-bond, G-bond, and 2D-bond, respectively. The relative intensity of the D-bond is generally related to the degree of disorder in the carbon layer structure, which reflects the lattice defects in the material to a certain extent. The G-bond is positively correlated with the degree of graphitization of the material. And the 2D-bond indicates the presence of a carbon layer inside the material. The *I*_D_/*I*_G_ of KAS_4-NVP and NVP samples are 1.037 and 0.984, respectively. This indicates that there are abundant defects on the surface of doped samples, which can provide more abundant diffusion channels and electrochemical active sites for the high-speed transport of ions and electrons during the charging–discharging process.^31,52,53^

**Figure 4:**
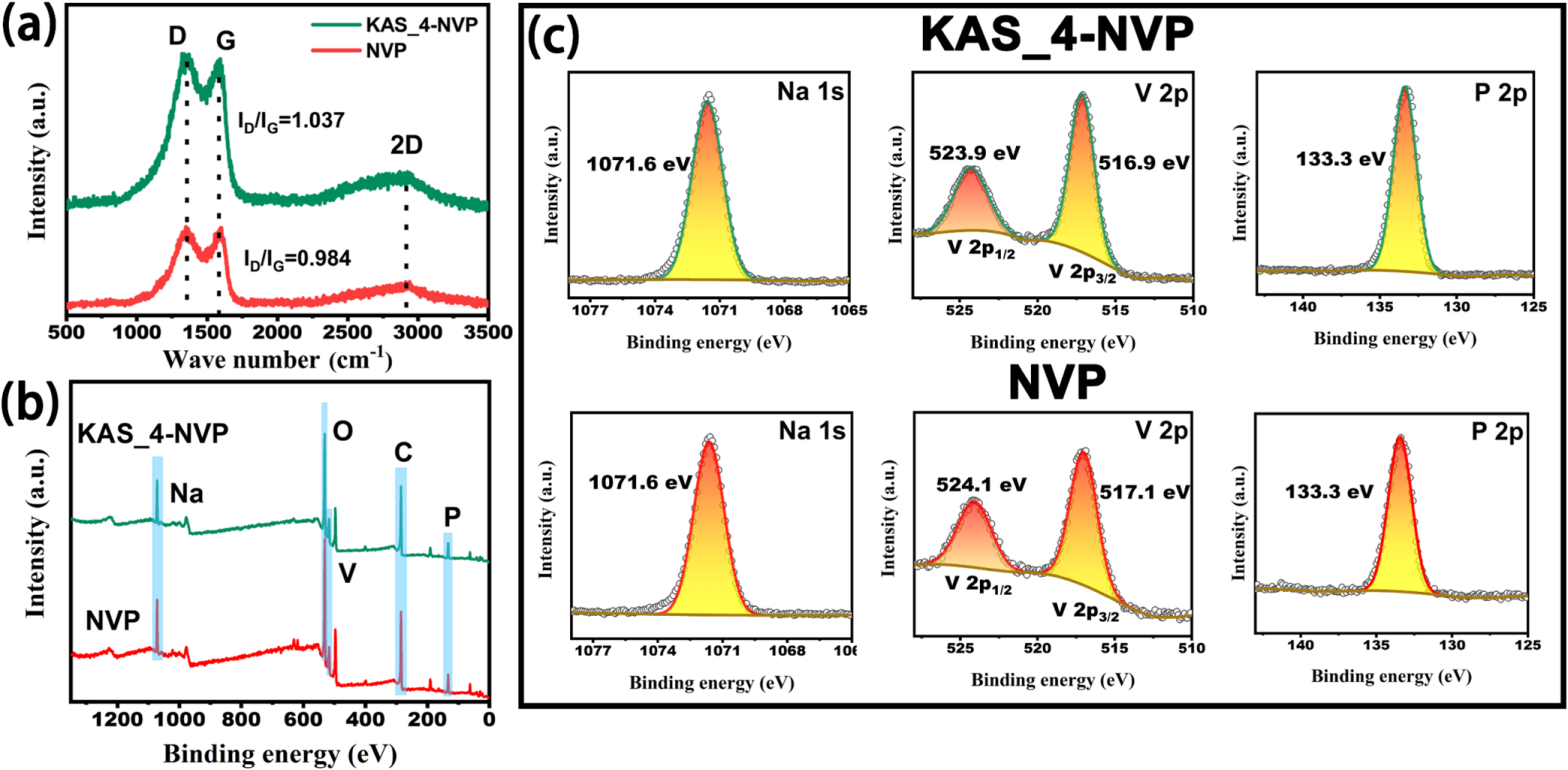
(a) Raman spectrum, (b) XPS survey spectrum, and (c) the high-resolution XPS spectra of Na 1s, V 2p, and P 2p for KAS_4-NVP and NVP sample.

In order to better analyze the chemical valence states of various elements in doped samples, XPS was carried out on the as-prepared samples. The XPS survey spectrum (Figure 4b) shows the chemical element composition of KAS_4-NVP and pure NVP. The signals of Na, V, P, O, and C can be clearly detected. By comparing the high-resolution XPS spectra of Na 1s, V 2p, and P 2p for the as-prepared sample (Figure 4c), it is not difficult to see that the binding energy of various elements in the doped sample is very close to that of pure NVP, which means that the chemical bonds of Na, V and P remain unchanged under the doping of low content K^+^, Al^3+^ and SO_4_^2–^ ternary ions. Although high-resolution XPS scans were attempted, the signal-to-noise ratio remained insufficient for a reliable analysis. However, due to the extremely low doping concentration of these guest species, the corresponding XPS signals of K, Al, and S are too weak to be reliably distinguished from the background noise in the survey spectra. This is a common limitation in XPS detection for trace dopants below the detection threshold. To confirm the successful incorporation of dopants, complementary inductively coupled plasma (ICP) analysis was performed, which provided quantitative elemental content and validated the presence of K^+^, Al^3+^, and SO_4_^2–^ in the doped samples (see Table S8).

To study the effect of multi-ion doping on the behavior of NVP during sodium storage, we carried out electrochemical tests on the KAS-NVP series cathode (Figure 5). The Cyclic voltammetry (CV) profiles of the KAS_4-NVP cathode at 0.2 mV·s^–1^ scanning rate are in the voltage range of 2.0–4.0 V (Figure 5a). It can be clearly seen that there is an obvious oxidation peak at 3.51 V, which corresponds to the V^3+^/V^4+^ redox reaction. However, the reduction peak of the KAS_4-NVP cathode splits into two distinct peaks at 3.32 and 3.23 V, respectively, during the discharge process. This is due to the rearrangement of the redox environment inside the material, which causes the diffusion of sodium ions from Na (1) to Na (2).^23,37^ In addition, the highly overlapping CV curves confirm the high reversibility and stability of the electrochemical reactions of the KAS_4-NVP cathode. The CV curves of other samples are shown in Figure S4, and their CV curves are basically the same as those of the KAS_4-NVP cathode, without significant changes.

**Figure 5:**
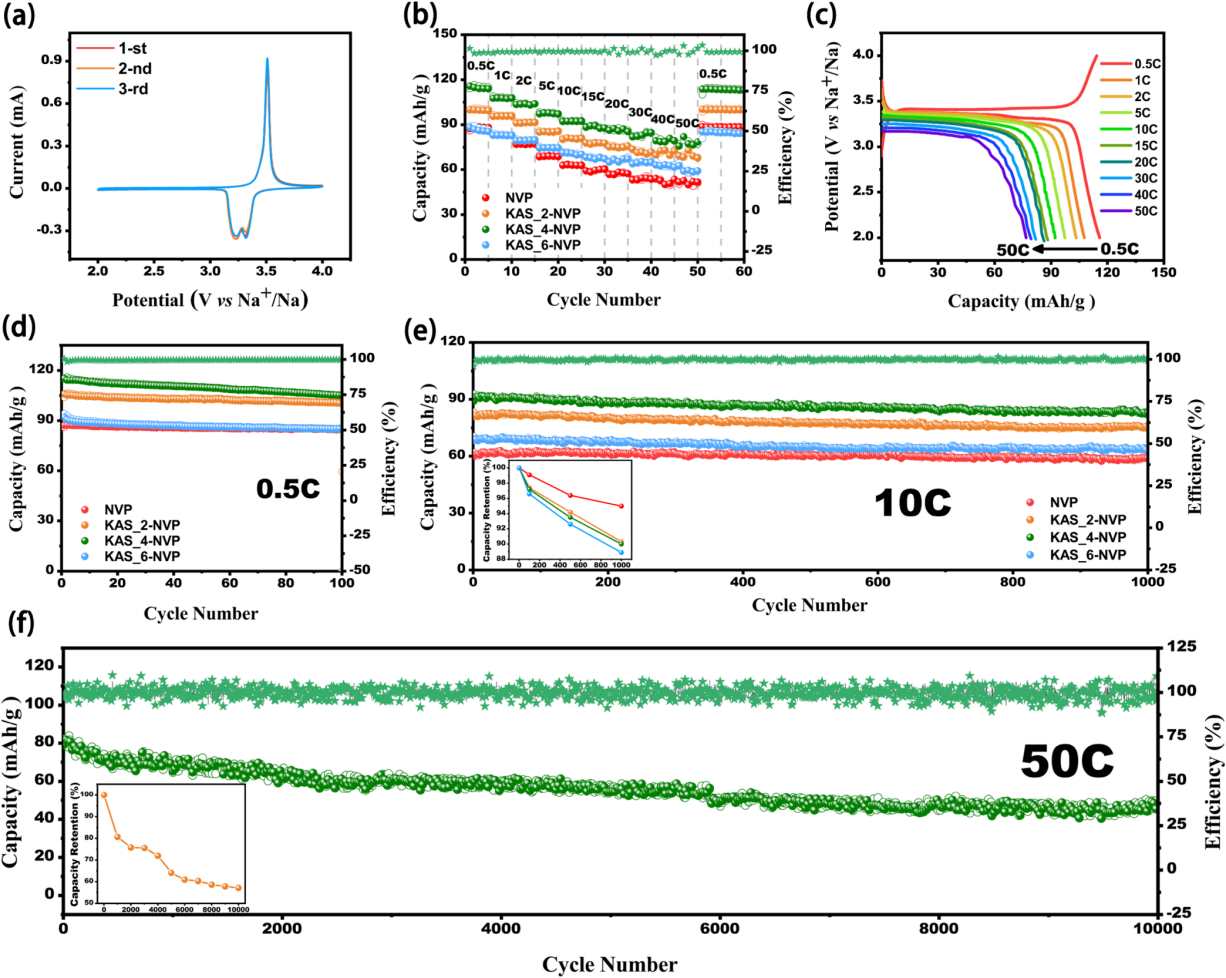
Electrochemical performance of Na_3–1.5*x*_K_0.5*x*_V_2–0.5*x*_Al_0.5*x*_(PO_4_)_3–*x*_(SO_4_)_*x*_/C (*x* = 0, 0.02, 0.04, 0.06) cathodes within the voltage window of 2.0–4.0 V versus Na^+^/Na: (a) Cyclic voltammetry profiles of KAS_4-NVP at 0.2 mV·s^–1^; (b) rate capability at different rates; (c) charging/discharging profiles of KAS_4-NVP cathode at different rates; (d) cycling stability at 0.5C; the long-term cycle performance of (e) as-prepared electrodes at a high rate of 10C and (f) KAS_4-NVP at a high rate of 50C (inset: Capacity retention, %).

In order to further evaluate the effect of multi-ion doping on the cycle stability and cycle life of NVP materials, we investigated the cycle behavior of as-prepared samples at different current densities. Figure 5b describes the rate performance from 0.5 to 50C of as-prepared cathodes (1C = 117 mA g^–1^). The discharge capacity of the NVP cathode is 87.4 mAh g^–1^ at 0.5C and decreases to 51.8 mAh g^–1^ when the current is 50C (the capacity retention rate at 50C is only 59.3%). Through comparison, it can be seen that the rate performance of K^+^, Al^3+^, and SO_4_^2–^ multi-ions doped samples is significantly superior to pure NVP. Among them, KAS_4-NVP cathode has the most excellent rate performance: at 0.5, 1, 2, 5, 10, 15, 20, 30, 40, and 50C are 115.9, 107.7, 103.5, 97.3, 92.2, 88.3, 86.3, 81.9, 79.4, and 75.8 mAh g^–1^, respectively. When the current returns to 0.5C again, a reversible capacity of 113.9 mAh g^–1^ can still be reached. Figures 5c and S5 describe the charge–discharge curves of the KAS_4-NVP and NVP cathodes at different rates. As presented in Figure 5c, the charge–discharge curves of the KAS_4-NVP cathode did not change significantly with the current increase from 0.5 to 50C. When the current density is 0.5C, there is a flat and slowly declining long platform around 3.37 V, corresponding to the V^3+/^V^4+^ redox reaction. It is consistent with the CV results. When the current increases to 10C, the voltage platform begins to show a slight downward shift, and when the current reaches 50C, the voltage platform shifts to 3.16 V, and its potential decreases by about 0.21 V.

The charge–discharge curves of NVP (Figure S5) are basically the same as that of the KAS_4-NVP cathode, but the electrochemical capacity is significantly lower than that of KAS_4-NVP at each rate. This means that multi-ion doping can effectively improve the lattice structure stability of NVP. The cycle stability of the KAS-NVP and S-NVP series cathodes at 0.5C is shown in Figure 5d and Table S9. In general, doping with low metal cation content can significantly enhance the electronic conductivity of NVP materials, thereby improving their electrochemical properties. Therefore, due to the doping of K^+^ and Al^3+^, the electrochemical performance of doped samples is generally higher than that of pure NVP. Among them, the KAS_4-NVP cathode exhibits the best electrochemical performance. The initial capacity of the KAS_4-NVP cathode can reach 115.3 mAh g^–1^ at 0.5C, which is close to the theoretical capacity of NVP (117.6 mAh·g^–1^). Moreover, the capacity retention rate reached as high as 91.3% after 100 cycles. Figure 5e and Table S10 exhibit the long-term cycle performance of as-prepared electrodes at a high rate of 10C. According to the electrochemical test results, the reversible capacity of the KAS_4-NVP cathode can still be maintained at 83.6 mAh g^–1^ after 1000 cycles, which is significantly higher than those of other as-prepared samples (62.7–75.2 mAh g^–1^) and pure NVP (58.9 mAh g^–1^). It should be noted that pure NVP shows the best long-term cycle stability in terms of capacity retention (inset in Figure 5e), which is significantly higher than the cycle performance of doped NVP at high rates reported in the past (Table S11). However, the capacity for undoped NVP still has the lowest value even after 1000 cycles. This may be because SO_4_^2–^ ions embedded in the phosphate skeleton structure of NVP significantly inhibit unwanted phase transitions, especially in the initial stage of charge and discharge. As a result of the synergistic effect of doping with several K^+^, Al^3+^, and SO_4_^2–^ ions, the KAS_4-NVP cathode shows a significantly better service life compared to undoped NVP. In order to further study the long cycle life of KAS_4-NVP cathode at a high rate, the ultralong cycle test (10,000 cycles at a high rate of 50C) was performed on KAS_4-NVP (Figure 5f). It is impressive that the KAS_4-NVP cathode can still achieve a high initial discharge capacity of 84.4 mAh g^–1^ at 50C. Moreover, the KAS_4-NVP cathode shows only 19.3 and 28% capacity decay after 1000 and 4000 cycles, and maintained a high capacity of 57.1% after 10,000 cycles (Table S12). This demonstrates the extraordinary ultralong cycle stability at the high rate of KAS_4-NVP cathode, which makes KAS_4-NVP cathode have broad application prospects.

The HR-TEM and XRD analyses were performed to investigate the structural evolution of the KAS4_NVP cathode after 500 cycles at different current rates (10 and 50C). HR-TEM images show that after cycling at 10C, the NVP particles largely maintain their crystallinity, with only minor and localized structural defects observed (highlighted by blue ovals). In addition, trace amounts of Na_3_PO_4_ impurities were detected within the thin carbon coating layer, along with signs of a nascent cathode–electrolyte interphase (CEI). These observations suggest that a stable, well-confined interfacial layer forms at moderate current, helping to preserve structural integrity and prolong cycling life (Figure S6a). In contrast, cycling at 50C results in severe amorphization, lattice collapse, and the formation of NaF secondary phases, indicating irreversible structural degradation (Figure S6b). The amorphous interfacial layer at this stage becomes significantly thicker and more disordered, which implies the growth of an unstable and heterogeneous CEI, along with the accumulation of inorganic decomposition products. Such changes disrupt ion transport and lead to a progressive impedance growth. These structural findings are further supported by XRD results, as presented in Figure S7. After 500 cycles at both 10C and 50C, the XRD patterns of the KAS_4-NVP cathode remain almost identical to the pristine material with well-preserved and sharp peaks characteristic of the NASICON-type structure. Only minimal broadening is observed, indicating that the bulk crystal structure remains stable, even during high-speed cycling. Together, the combined HR-TEM and XRD analyses demonstrate that KAS4_NVP retains structural stability under moderate–rate cycling due to limited CEI growth and interfacial integrity. However, under high-rate cycling (50C), excessive interfacial reactions and CEI thickening induce bulk degradation and capacity fading, highlighting the critical role of interface stability for long-term cycling performance.

In order to further determine the reason why ternary ion doping improves the electrochemical performance of the KAS_4-NVP cathode, electrode reaction kinetics was performed. Figure 6a shows the galvanostatic intermittent titration technique (GITT) profile of the KAS_4-NVP cathode during the second cycle of charge and discharge, reflecting the estimated diffusion coefficient of sodium ions *D*_Na^+^_ (the battery is charged and discharged at 0.1C for 30 min within a voltage window of 2.0–4.0 V, and then rests for 120 min to reach a balanced state). The GITT curve parameters are shown in Figure S8a, and the GITT results of other samples are shown in Figure S8b–d. As can be seen, the *D*_Na^+^_ of the KAS_4-NVP cathode is about 10^–13^–10^–10^ cm^2^ s^–1^, while that of pure NVP is approximately in the range of 10^–14^–10^–10^ cm^2^ s^–1^. Must be noted that the synergistic effect of ternary ion doping contributes to the enhancement of the sodium-ion diffusion kinetics of the NVP battery system, which is one of the key factors for the extraordinary rate performance of the KAS_4-NVP cathode. Importantly, these experimental findings are well supported by bond valence site energy (BVSE) calculations. The calculated Na^+^ migration energy barrier for KAS_4_–NVP is 0.369 eV, markedly lower than that of the undoped NVP (0.434 eV), indicating a ∼15% reduction in the activation barrier. The close agreement between the decreased migration energy and the order-of-magnitude increases in *D*_Na^+^_ derived from GITT measurements substantiates the reliability of the BVSE model and confirms that codoping effectively facilitates Na^+^ transport. Such consistency between the theoretical and experimental results underscores the improved ionic conductivity and enhanced electrochemical kinetics in the doped NVP cathode system.

**Figure 6:**
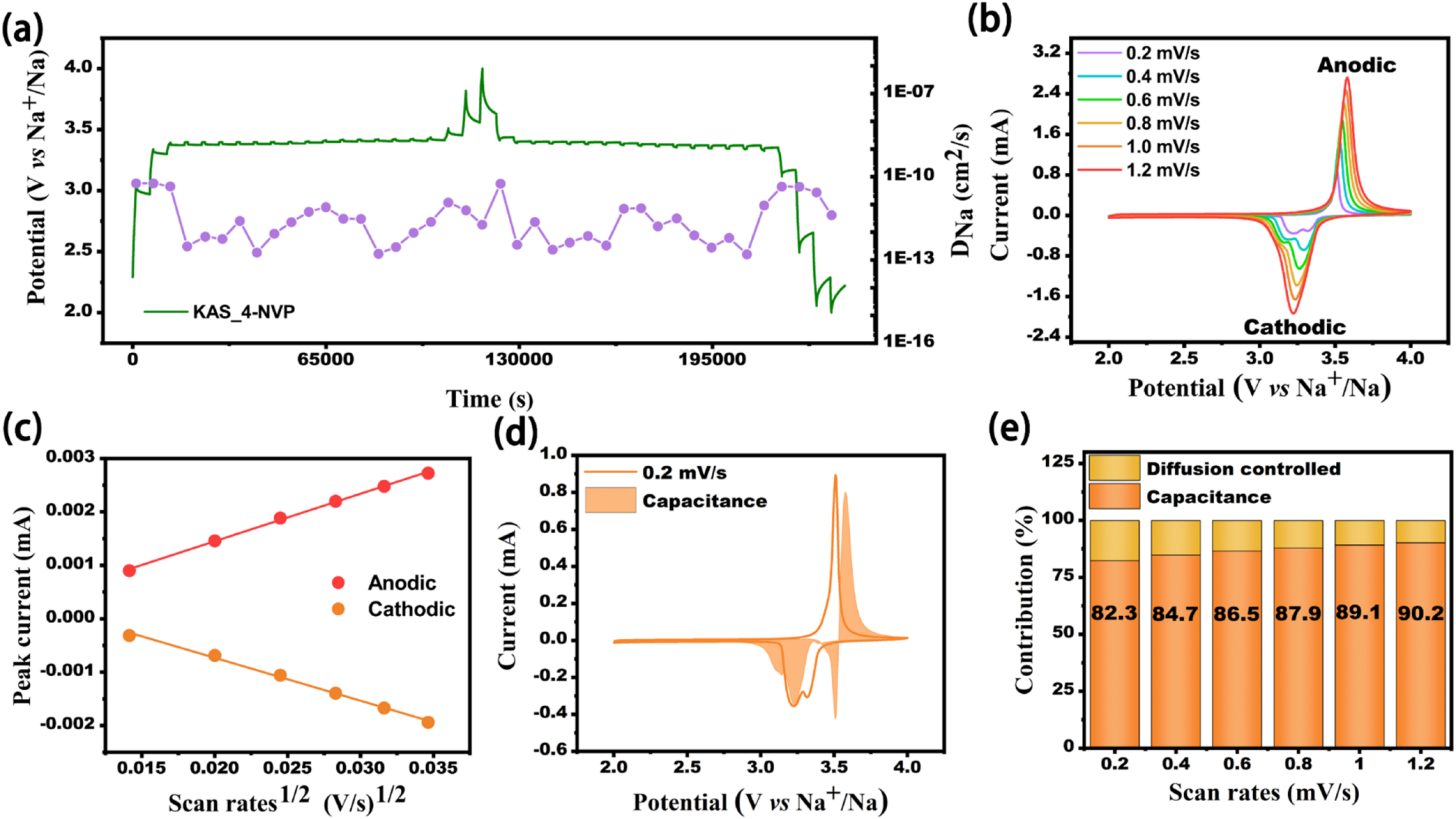
Kinetic properties of KAS_4-NVP cathode: (a) The Galvanostatic intermittent titration technique (GITT) profile and the corresponding calculated Na^+^ diffusion coefficients (*D*_Na^+^_); (b) CV curves at various scan rates from 0.2 to 1.2 mV s^–1^; (c) linear relationships between the square root of scan rate and the peak current; (d) the calculated capacitance at 0.2 mV s^–1^; and (e) pseudocapacitive contribution at different scan.

In addition, CV curves at various scan rates were performed for the KAS_4-NVP cathode in the voltage range 2.0–4.0 V to further analyze the kinetic characteristics of electrodes (Figure 6b). The current peaks of the fitted oxidation and reduction peaks exhibit a linear relationship with the square root of the scanning rate (Figure 6c). According to the Randles Sevcik equation (see detailed in Supporting Information), the values of the sodium-ion diffusion coefficient *D*_Na^+^_ can be approximated, about 1.47 × 10^–11^ (Anodic) and 1.20 × 10^–11^ (Cathodic), which is consistent with the GITT results. In general, the sodium storage methods of carbon-coated electrode materials in SIBs include non-Faraday (surface diffusion control) and Faraday (pseudocapacitance) processes. Among them, pseudocapacitance contribution is one of the important indicators used to evaluate the reaction kinetics of the electrode interface, playing a crucial role in the rate performance and cycled life of electrode materials. Therefore, the relevant calculations on the pseudocapacitance contribution of the KAS_4-NVP cathode were performed (detailed in SI). Through calculation, it can be seen that the pseudocapacitance contribution is approximately 82.3% of the total current at 0.2 mV s^–1^ (shaded part of Figure 6d). It is worth noting that as the scanning rate increases, the pseudocapacitance contribution also increases, eventually reaching a maximum value of 93.4% at 1.2 mV s^–1^ (Figure 6e). The excellent pseudocapacitance contribution is one of the important factors of the KAS_4-NVP cathode with extraordinary long cycle life at a high rate.

Moreover, electrochemical impedance spectroscopy (EIS) of the KAS-NVP series samples was performed (Figure S9 and Table S13). Nyquist graphs for all prepared electrodes charged to 4 V after CV at 0.2 mV s^–1^ are shown in Figure S9a (detailed in SI). The equivalent circuit for fitting the EIS shown in the illustration in Figure S7a facilitates further analysis of the electrode kinetic parameters of the as-prepared samples. The kinetic parameters of all samples fitted according to the selected equivalent circuit diagram are shown in Table S13. The contact resistance at the interface between electrode and electrolyte *R*_s_ values is similar, while the charge transfer resistance *R*_p_ values for doped samples are significantly less than NVP, and the *R*_p_ of the KAS_4-NVP sample is the smallest (100.2 Ω). According to the Warburg impedance in the low-frequency range of the EIS, the sodium-ion diffusion coefficients (*D*_Na^+^_) were calculated (detailed in SI), and the relationship *Z*′ and ω-1/2 are displayed in Figure S9b. Among them, KAS_4-NVP has the highest *D*_Na^+^_ (5.56 × 10^–12^ cm^2^ s^–1^), and this is consistent with the results of the GITT test and CV test at different scan rates, as described above. However, the *D*_Na^+^_ of pure NVP is 6.58 × 10^–13^ cm^2^ s^–1^, which is significantly lower than the synergistic effect of ternary ion codoping and can significantly enhance the electrode kinetic parameters and sodium-ion diffusion coefficient of the NVP cathode.

In addition, the comparison table of the discharge capacity and cycle life of as-prepared KAS-NVP and S-NVP samples with similar single-ion or multi-ions doped NVP cathodes (Table S10). It can be clearly seen that the electrochemical properties of the KAS_4-NVP cathode are significantly superior to or similar to those previously reported. It is worth noting that the improvement of electrochemical performance and kinetic characteristics of KAS-NVP series samples is attributed to the synergistic effect of K^+^, Al^3+^, and SO_4_^2–^ multi-ions doping rather than the individual action of a single ion. For the doping of metal cations, many studies have shown that the sole doping of Al^3+^ and K^+^ can improve the electronic conductivity of NVP and enhance the stability of the lattice structure, thus improving its electrochemical capacity and cycle performance at a low current density. However, in terms of high-rate performance and long cycle life, Al-NVP^29,36^ and K-NVP^26^ still have a certain gap compared with the KAS_4-NVP cathode. For the doping of anions, the doping of the SO_4_^2–^ ion on the PO_4_^3–^-site in NVP has not been reported in the literature so far.

Figure S10 describes the cyclic behavior of S-NVP at different currents (voltage ranges of 2.0–4.0 V). The cycle stability of S-NVP series cathodes at 0.5C is shown in Figure S10a and Table S9. It can be seen that the initial capacity of doped samples and pure NVP at a low current of 0.5C is not much different, about 85–90 mAh g^–1^. So, doping SO_4_^2–^ ion does not significantly improve the electrochemical capacity of the NVP cathode. However, with the increase of current, the rate performance of S-NVP cathodes is improved to varying degrees (Figure S10b), and the S4-NVP cathode has the best rate performance. The discharge capacity of S4-NVP at 0.5, 1, 2, 5, 10, 15, 20, 30, 40, and 50C are 91.2, 88.5, 86.2, 83.5, 81.2, 78.1, 77.8, 75, 74.8, and 73.9 mAh g^–1^, respectively. The capacity retention rate at 50C is about 81%, and the capacity can reach 89.2 mAh g^–1^ when it returns to 0.5C. Moreover, S-NVP cathodes also exhibit an excellent long cycle life (Figure S10c and Table S10). According to the electrochemical test results, after 1000 cycles of charge and discharge, the S4-NVP cathode still has a reversible capacity of 73.9 mAh g^–1^, and the capacity retention rate is 86.9%. Although SO_4_^2–^ is an anionic group and does not significantly enhance the electronic conductivity of the NVP cathode, both the rate capability and cycling stability are improved due to its incorporation into the NASICON-type phosphate framework. Since both the undoped and SO_4_^2–^-doped samples possess similar carbon coatings, the performance enhancement is primarily attributed to SO_4_^2–^substitution, which modifies the local crystal environment, stabilizes the lattice structure, and facilitates Na^+^ transport during repeated cycling. However, there is still a gap between the improved electrochemical performance of the S-NVP cathode and that of the KAS_4-NVP cathode. At the same time, we also analyzed and studied the electrochemical impedance spectra (EIS) of the S-NVP series samples, and the results are shown in Figure S11 (the kinetic parameters obtained by fitting the EIS data are presented in Table S13). It can be clearly seen that the conductivity and diffusion coefficient of S-NVP samples are significantly higher than that of pure NVP. The S4-NVP cathode has the smallest charge transfer resistance *R*_p_ (152.5 Ω) and the highest sodium diffusion coefficient *D*_Na^+^_ (3.86 × 10^–12^ cm^2^ s^–1^). However, it is still smaller than the KAS_4-NVP cathode (*R*_p_ = 100.2 Ω, *D*_Na^+^_ = 5.56 × 10^–12^ cm^2^ s^–1^). Thus, the excellent high-rate characteristics and long life of the KAS_4-NVP cathode are mainly due to the synergistic effect of K^+^, Al^3+^, and SO_4_^2–^ ions codoping.

In order to further study the electrochemical sodium storage mechanism of the KAS_4-NVP cathode material, in situ XRD was used to analyze the two-phase transition process of the KAS_4-NVP cathode during charge and discharge (Figure 7). A special battery with the window and carbon cloth as the collector was assembled to observe the in situ reaction during the first cycle of charge and discharge. In general, the reversible changes of some diffraction peaks during the charge–discharge process can be clearly observed by the in situ XRD approach, which also means that the electrochemical reaction is highly reversible. In Figure 7, it can be seen that in the initial cycle, within a specific 2θ range, the diffraction peak of the typical nanostructure with the *R*3̅*c* space group can be observed. During the charge-discharging process in the voltage range of 2.0–4.0 V, the separation of (104) reflection and the reversible disappearance/emergence of peak reflections (211/116) exhibits a typical two-phase transformation.^54^ In addition, the diffraction peaks corresponding to the (110), (113), (024) and (300) reflections experience a reversible continuous left–right shift, which is attributed to the existence of a solid-solution reaction.^54,55^ The reversible shift of these diffraction peaks is due to the shrinkage and reduction of the lattice framework caused by the occupation of Na atoms in the redox reaction of transition metals in the [MO_6_] octahedron accompanied by the Na^+^ ion extraction/insertion.^54,55^ The results show that the solid NASICON frame structure is one of the important guarantees for the reversible evolution of the stable lattice structure of the KAS_4-NVP cathode in the charge-discharging process.

**Figure 7:**
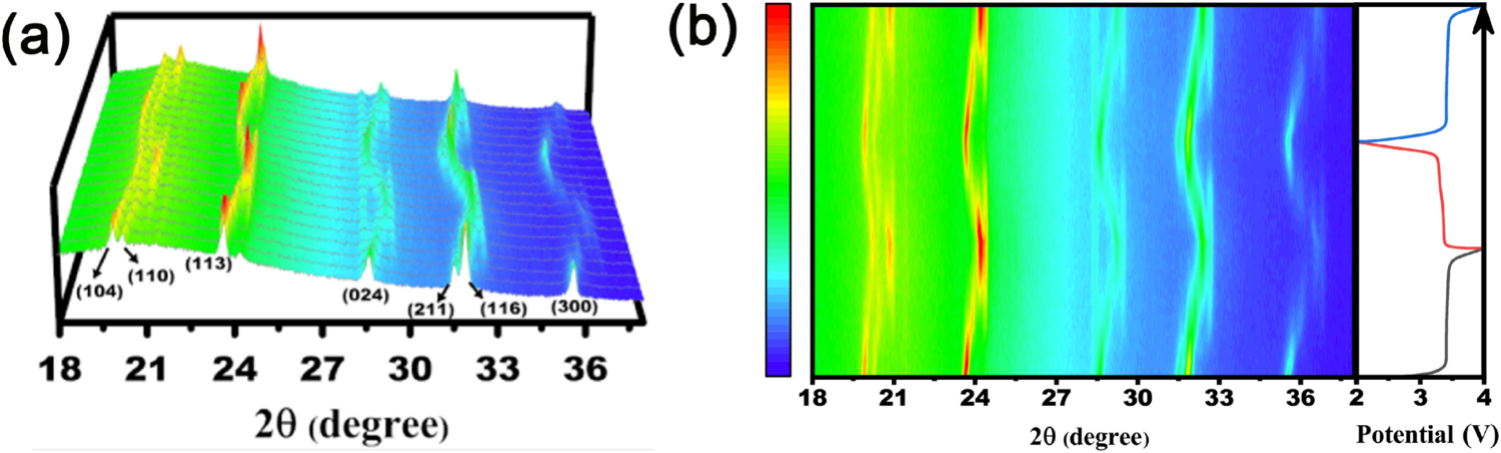
Operando XRD patterns for the KAS_4-NVP electrode: (a) 3D map of operando XRD data and (b) the contour map.

The KAS_4-NVP cathode demonstrates significant potential for wide application prospects due to its excellent rate performance and cycle stability. Considering energy density, price cost, application field, and other factors, sodium-free-anode sodium metal batteries (SFA-SMBs) stand out among many choices.^56–59^ The SFA-SMBs comprise a cathode, an anodic current collector, separator, and electrolyte. Its working principle relies on the in situ deposition and stripping of sodium ions released from cathode materials during the charge–discharge process on the surface of the anode current collector.^60–62^ Therefore, their energy density, operating voltage, electrochemical capacity, and other electrochemical properties mainly depend on the cathode materials. In this work, KAS_4-NVP cathode, Cu/SP foil (anodic current collector), a double-layered separator consisting of a Celgard 2500 film and a Whatman GF/F glass fiber, and electrolyte (1.0 M NaPF_6_ in diglyme = 100 Vol %) are used to assemble the KAS_4-NVP||Cu/SP SFA-SMBs.

Before the KAS_4-NVP||Cu/SP SFA-SMBs assembly, the KAS_4-NVP cathode is predischarged (discharged to 0.05 V at 0.5C), and the charge–discharge curve is shown in Figure S12. During the discharge process, V^3+^ is partially reduced to V^2+^, which causes Na^+^ from the electrolyte to the KAS_4-NVP lattice.^45,63,64^ As a result, the conversion of Na_2.94_K_0.02_V_1.98_Al_0.02_(PO_4_)_2.96_(SO_4_)_0.04_ to Na_3.94_K_0.02_V_1.98_Al_0.02_(PO_4_)_2.96_(SO_4_)_0.04_ takes place (cathode is in a sodium-rich state, and the excess sodium can make up for the capacity loss in the first cycle of the SFA-SMBs^58^). In addition, we assembled Cu/SP and Cu into the Cu/SP||Na and Cu||Na cells and compared their electrochemical properties (Figure S13 and detailed in the SI). The test results show that Cu/SP foil has a small nucleation overpotential, which can significantly improve the cycle stability of symmetric batteries, and help Na^+^ stable in situ deposition and stripping on the collector during charge and discharge.^60,61^

The electrochemical performance of KAS_4-NVP||Cu/SP SFA-SMBs is shown in Figure 8. The charge and discharge curve and cycle stability of the SFA-SMBs in the voltage range of 2.0–3.8 V at 0.5C. Since KAS_4-NVP is predischarged to 0.05 V, the open circuit voltage of SFA-SMBs is less than 0 V after assembly (Figure 8a,b). During the first charging process, there is an additional platform at 1.63 V relative Na/Na^+^, corresponding to the extraction of the presodiated Na from the Na_3.94_K_0.02_V_1.98_Al_0.02_(PO_4_)_2.96_(SO_4_)_0.04_/C electrode. The initial capacity of the KAS_4-NVP cathode reaches 221.7 mAh g^–1^, while the initial discharge capacity is 82.1 mAh g^–1^, which is largely attributed to the destruction of the electrolyte at the cathode side as well as the sodiation of the carbon layer and the formation of the SEI.^58^ However, after 100 cycles in the voltage range 2.0–3.8 V, the SFA-SMBs still have a high reversible capacity of 80.6 mAh g^–1^, the capacity retention rate is as high as 98.2%, and its operating voltage is almost not reduced. The rate performance of the SFA battery is shown in Figure 8c. The discharge capacity of KAS_4-NVP||Cu/SP SFA-SMBs at 0.5, 1, 2, 3, 5, and 10C are 83.2, 78.0, 74.0, 71.6, 68.9, and 62.2 mAh g^–1^, respectively. And the capacity can still reach 81.5 mAh g^–1^ after returning to 0.5C. This shows that KAS_4-NVP||Cu/SP SFA-SMBs have excellent rate performance. Moreover, the long-term cycle performance of KAS_4-NVP||Cu/SP SFA-SMBs at a high rate of 5C is shown in Figure 8d. The initial capacity of the SFA battery at 5C is 69.2 mAh g^–1^, the capacity retention reaches 88.4% after 700 cycles, and the Coulomb efficiency is always greater than 97%. It can be seen that KAS_4-NVP||Cu/SP SFA-SMBs have excellent electrochemical performance at different rates, which is mainly attributed to the ultrafast Na insertion/extraction kinetics in the NASICON-structured cathode and rapid Na plating/stripping dynamics at the anodic current collector. It is well established that the first-cycle Coulombic efficiency and cycling lifespan of SFA-SMBs are primarily constrained by irreversible sodium loss stemming from “dead Na” formation on the anode current collector surface, alongside uncontrolled Na dendrite growth during cycling. The nonuniform deposition of sodium or volume fluctuations during the deposition/stripping processes disrupt the SEI layer, causing continuous electrolyte consumption and exacerbating dead Na formation. This, in turn, leads to reduced cycling stability, deteriorated Coulombic efficiency, and even penetration of Na dendrites through the separator, causing short circuits. To enhance the performance of SFA-SMBs, a dual approach is essential. On the anode side, the key strategy involves constructing and modifying a metallic sodium nucleation layer on the current collector. This modification promotes uniform sodium deposition/stripping, suppresses dendrite growth, and thereby improves the electrochemical cycling stability. On the cathode side, a presodiation strategy to pre store a large amount of Na in NASICON-structured cathodes with stable structures can be employed. In this work, the prestored sodium atoms in KAS_4-NVP effectively compensate for sodium loss during cycling. Furthermore, this material enables a reversible vanadium-based two-electron redox reaction within a voltage window of 2.0–3.8 V voltage window. And the Cu/SP foil significantly enhances uniform sodium deposition/stripping at the anode. Consequently, the KAS_4-NVP||Cu/SP SFA-SMBs configuration delivers remarkable electrochemical performance, including a high specific capacity and prolonged cycle life.

**Figure 8:**
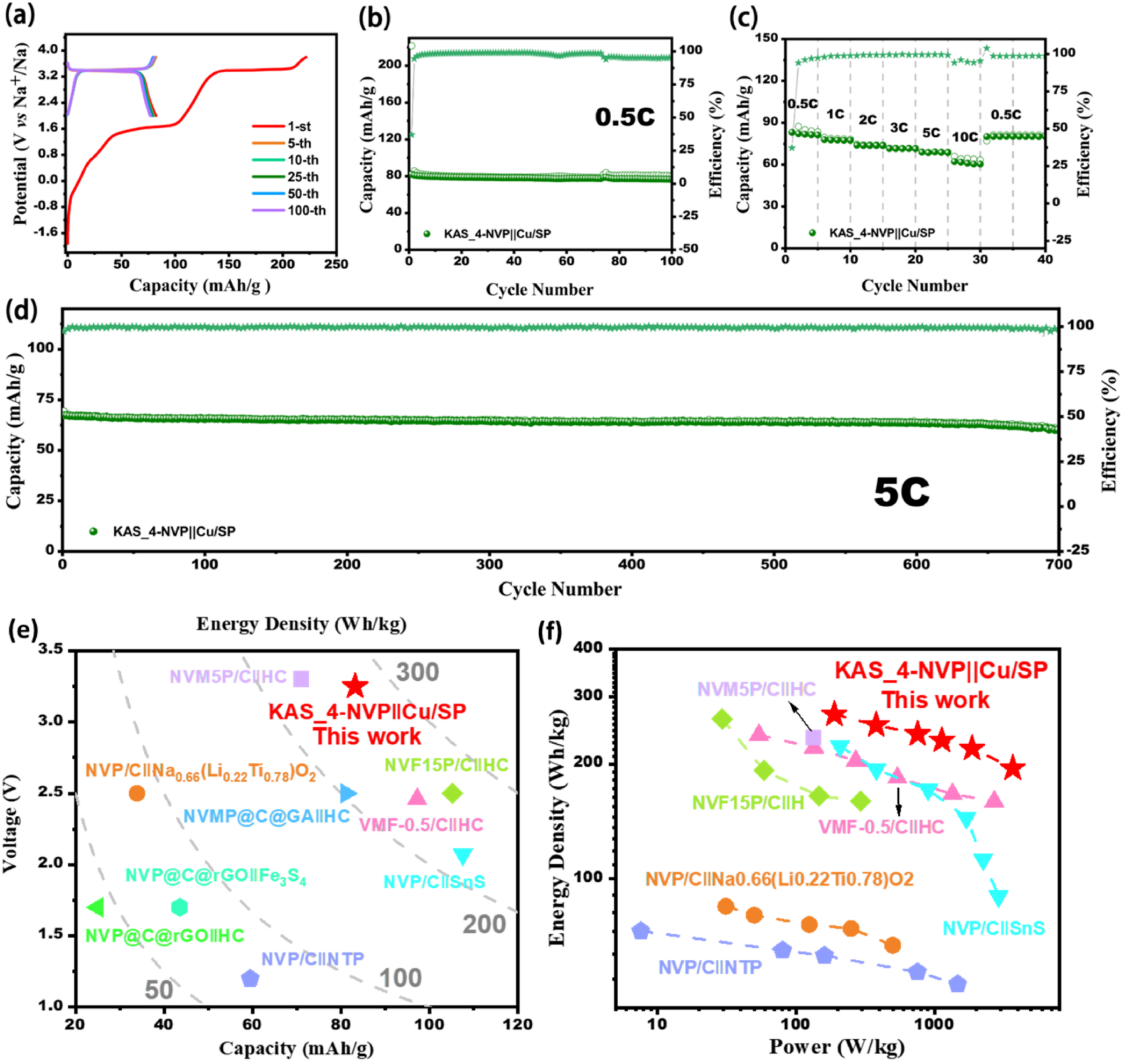
Electrochemical performance of KAS_4-NVP||Cu/SP cells within the voltage window of 2.0–3.8 V versus Na^+^/Na: (a) Charging/discharging profiles and (b) cycling stability at 0.5C; (c) rate capability at different rates; (d) long-term cycle performance at a high rate of 10C. The comparison of voltage and capacity (e) and Ragone plots comparing (f) for the KAS_4-NVP||Cu/SP SFA-SMBs with other state-of-the-art reported sodium-ion full-cell configurations. The energy and power densities are based on the total mass of the cathode and anode active materials.

In addition, the KAS_4-NVP||Cu/SP SFA-SMBs exhibit significantly superior energy and power densities compared to those of state-of-the-art sodium-ion full cells (Figure 8e,f). The assembled KAS_4-NVP||Cu/SP SFA-SMBs deliver an average operating voltage of 3.25 V and achieve a high energy density of 270.1 Wh kg^–1^ (Figure 8e). The data presented in Figure 8f clearly demonstrate their outstanding advantages in both energy and power density over other sodium-ion full-cell configurations reported in the literature.^65–72^

## Conclusions

In this study, a series of novel NASICON-type cathode materials, Na_3–1.5*x*_K_0.5*x*_V_2–0.5*x*_Al_0.5*x*_(PO_4_)_3–*x*_(SO_4_)_*x*_/C, were designed and synthesized by multi-ion doping at various sites within the NVP structure via a sol–gel method, incorporating inexpensive alum. The XRD Rietveld refinement and XPS confirmed the successful embedding of K^+^, Al^3+^, and SO_4_^2–^ into the NASICON-type crystal structure. Ternary doping has greatly improved the performance and durability of the electrode in sodium-ion batteries (SIBs). The developed cathode exhibits outstanding high-rate electrochemical and kinetic characteristics. The KAS_4-NVP cathode has a high reversible capacity of 115.3 mAh g^–1^ at 0.5C, and still has a high initial capacity of 79.1 mAh g^–1^ at 50C. Furthermore, SFA-SMBs (sodium full cells) composed of the KAS_4-NVP cathode paired with Cu/SP foil demonstrated excellent long-term cycling stability. This research represents a significant advancement toward developing next-generation sodium-ion batteries, highlighting the potential of doped NASICON-type materials for high-performance energy storage applications. Therefore, the principles of multisite doping can become an excellent basis for further expanding the possibilities of the electrochemical characteristics of such systems.

## Experimental Section

### Sample Preparation

Synthesis of the Na_3–1.5*x*_K_0.5*x*_V_2–0.5*x*_Al_0.5*x*_(PO_4_)_3–*x*_(SO_4_)_*x*_ material: The Na_3–1.5*x*_K_0.5*x*_V_2–0.5*x*_Al_0.5*x*_(PO_4_)_3–*x*_(SO_4_)_*x*_/C (*x* = 0, 0.02, 0.04, 0.06) cathodes were synthesized by the sol–gel method using Na_2_CO_3_, NH_4_VO_3_, KAl­(SO_4_)_2_·12H_2_O, NH_4_H_2_PO_4_, and citric acid as raw materials (all reagents are of analytical grade) according to chemical eq 1

1 $$\left(3-1.5_{x}\right){\mathrm{Na}}_{2}{\mathrm{CO}}_{3}+\left(4-x\right){\mathrm{NH}}_{4}{\mathrm{VO}}_{3}+xKAI{\left({\mathrm{SO}}_{4}\right)}_{2}+\left(6-2x\right){\mathrm{NH}}_{4}H_{2}{\mathrm{PO}}_{4}\to 2{\mathrm{Na}}_{3-1.5x}K_{0.5x}V_{2-0.5x}{\mathrm{AI}}_{0.5x}{\left({\mathrm{PO}}_{4}\right)}_{3-x}{\left({\mathrm{SO}}_{4}\right)}_{x}+\left(3-1.5x\right){\mathrm{CO}}_{2}+\left(10-3x\right){\mathrm{NH}}_{3}+\left(5-1.5x\right)H_{2}O$$

To synthesize Na_3–1.5*x*_K_0.5*x*_V_2–0.5*x*_Al_0.5*x*_(PO_4_)_3–*x*_(SO_4_)_*x*_ cathodes (Figure 1), first, NH4VO3, and citric acid were dissolved in deionized water to obtain a yellow solution. After heating and stirring at 80 °C for 0.5 h, the solution turns yellow to clear blue. Then KAl­(SO_4_)_2_·12H_2_O, NH_4_H_2_PO_4_, and Na_2_CO_3_ in the required stoichiometric amounts were successively added to the solution and continued to stir at 80 °C until a large amount of water was evaporated and the gel formed. The as-prepared gel was dried in the drying oven at 80 °C for 12 h, and then dried at 120 °C for 3 h. The as-prepared powder was calcined in the argon atmosphere at 350 and 750 °C for 4 and 8 h, respectively. And the NASICON-type Na_3–1.5*x*_K_0.5*x*_V_2–0.5*x*_Al_0.5*x*_(PO_4_)_3–*x*_(SO_4_)_*x*_ cathodes were obtained. Hereinafter, for simplification, we abbreviate the designation of samples of Na_3–1.5*x*_K_0.5*x*_V_2–0.5*x*_Al_0.5*x*_(PO_4_)_3–*x*_(SO_4_)_*x*_ as KAS-NVP, while the Na_3–1.5*x*_K_0.5*x*_V_2–0.5*x*_Al_0.5*x*_(PO_4_)_3–*x*_(SO_4_)_*x*_ (*x* = 0, 0.02, 0.04, 0.06) are denoted as NVP, KAS_2-NVP, KAS_4-NVP, and KAS_6-NVP, respectively.

Synthesis of the Na_3–*x*_V_2_(PO_4_)_3–*x*_(SO_4_)_*x*_/C material: The Na_3–*x*_V_2_(PO_4_)_3–*x*_(SO_4_)_*x*_/C (*x* = 0.02, 0.04, 0.06) cathodes were synthesized by the sol–gel method using Na_2_CO_3_, NH_4_VO_3_, Na_2_SO_4_, NH_4_H_2_PO_4_ and citric acid as raw materials (all reagents are of analytical grade) according to chemical eq 2

2 $$\left(3-3x\right){\mathrm{Na}}_{2}{\mathrm{CO}}_{3}+4{\mathrm{NH}}_{4}{\mathrm{VO}}_{3}+2{xNa}_{2}{\mathrm{SO}}_{4}+\left(6-2x\right){\mathrm{NH}}_{4}H_{2}{\mathrm{PO}}_{4}\to 2{\mathrm{Na}}_{3-x}V_{2}{\left({\mathrm{PO}}_{4}\right)}_{3-x}{\left({\mathrm{SO}}_{4}\right)}_{x}+\left(3-3x\right){\mathrm{CO}}_{2}+\left(10-2x\right){\mathrm{NH}}_{3}+\left(5+x\right)H_{2}O$$

The synthesis method for Na_3–*x*_V_2_(PO_4_)_3–*x*_(SO_4_)*_x_*/C is the same as that of the KAS-NVP cathode. Similarly, in the following article, for simplicity, we denote the designed Na_3–*x*_V_2_(PO_4_)_3–*x*_(SO_4_)_*x*_/C as S-NVP, while the Na_3–*x*_V_2_(PO_4_)_3–*x*_(SO_4_)_*x*_/C (*x* = 0.02, 0.04, 0.06) are denoted as S2-NVP, S4-NVP and S6-NVP, respectively.

### Material Characterization

The X-ray diffraction (XRD) patterns of the obtained samples were collected at room temperature with powder diffractometers RIGAKU, D/MAX 2550 V, and DX-2700B (Cu Kα radiation λ = 1.54056 Å). Rietveld refinement was made using GSAS II software.^73^ The microstructure of KAS-NVP and S-NVP materials was characterized by using field-emission scanning electron microscopy (FESEM, Magellan 400) with an Oxford X-Max microanalysis EDS system after sputtering with Au. High-resolution transmission electron microscopy (HR-TEM) images were collected on a field-emission JEOL JSM–2010F. Raman spectra were measured by using Raman spectroscopy (Renishaw) with 532 nm laser excitation. The surface chemical states of samples were investigated utilizing X-ray photoelectron spectroscopy (XPS) performed on an ESCALAB 250 electron spectrometer.

### Electrochemical Tests

#### Preparation of Cathode Electrodes for KAS-NVP and S-NVP

The electrode slurry is prepared by mixing the active material, carbon black, and PVDF in the *N*-methylpyrrolidone (NMP) with a mass ratio of 7.5:2.0:0.5. The as-prepared slurry is coated on Al foil and dried in a vacuum drying oven at 110 °C for 12 h. Then a cathode electrode with a thickness of 120 μm was obtained. The obtained cathode is cut into a circular electrode piece with a diameter of 12 mm. The load mass of the active material on the electrode is 1.05–2.025 mg cm^–2^. For the half battery, the CR2032 coin cell is assembled in a glovebox filled with Ar. The coin cell is assembled from the prepared cathode, metal sodium (the anode), Whatman GF/F glass fiber separator, and 60 uL of electrolyte (1 M NaClO_4_ in a mixture of ethyl carbonate (EC) and dimethyl carbonate (DEC) = 1:1 vol % with 5.0% fluoroethylene carbonate (FEC)). After standing the prepared cell for 12 h, the LAND CT2001A battery test system is used to perform the constant current charge–discharge experiment and GITT in the voltage window of 2.0–4.0 V, where 1C = 117 mA g^–1^. The electrochemical impedance spectroscopy (EIS) and cyclic voltammetry (CV) tests at various scan rates from 0.2 to 1.2 mV/s were applied using a CHI-760E electrochemical workstation CHI-760E (China). The EIS was performed in the frequency range from 10 MHz to 0.01 Hz with an amplitude of 10 mV at the open circuit potential. ZView software was used to simulate the equivalent circuit of EIS and calculate the parameters of the equivalent circuit.

#### Preparation of Cu/SP Foil

Super P and PVDF are evenly mixed in the *N*-methylpyrrolidone (NMP) with a mass ratio of 9:1, then coated on Cu foil, and dried at 110 °C in the vacuum drying oven for 12 h to obtain Cu/SP foil. The prepared Cu/SP foil is cut into a circular electrode piece with a diameter of 16 mm. The load mass of the SP layer on the electrode is 0.1–0.2 mg cm^–2^. To evaluate Na plating/stripping efficiencies, CR2032-type coin cells were assembled inside the glovebox using Cu/SP or Cu as the working electrode, Na metal as the reference electrode with a double-layered separator consisting of a Celgard 2500 film and a Whatman GF/F glass fiber, and electrolyte (1.0 M NaPF_6_ in diglyme = 100 Vol %). 0.5 mAh cm^–2^ portion of Na was deposited on the collectors at different areal currents and recharged to 0.3 V to strip the deposited Na. The CE is defined as the ratio of stripped capacity to plated capacity.

#### Preparation of KAS_4-NVP||Cu/SP SFA-SMBs

The SFA-SMBs are composed of a KAS_4-NVP cathode, an anodic current collector (Cu/SP foil), a double-layered separator consisting of a Celgard 2500 film and a Whatman GF/F glass fiber, and electrolyte (1.0 M NaPF6 in diglyme = 100 Vol %). Among them, the active material load mass of the KAS_4-NVP cathode is 4–7 mg. After standing the prepared cell for 12 h, the LAND CT2001A battery test system performs the constant current charge–discharge experiment and GITT in the voltage window of 2.0–4.0 V, where 1C = 117 mA g^–1^.

### Computational Details

Bond valence site energy (BVSE) Calculations used to investigate the migration of Na ions are based on the SoftBV program, using the single crystal structural model as input and the SoftBV bond valence parameter set developed by S. Adams.^47,48^ Sodium-ion site energies were calculated for a dense grid of points with a resolution of 0.1 Å covering the crystal structure by using the transferable Morse-type softBV force field. Na-ion diffusion pathways are identified with the regions with a low bond valence site energy. Bond valence site energy (BVSE) Calculations used to investigate the migration of Na ions are based on the SoftBV program, using the single crystal structural model as input and the SoftBV bond valence parameter set developed by S. Adams. Sodium-ion site energies were calculated for a dense grid of points with a resolution of 0.1 Å covering the crystal structure using the transferable Morse-type softBV force field. Na-ion diffusion pathways are identified with the regions of low bond valence site energy. The KAS_4-NVP and NVP structures determined from XRD were used as the input structures for the BVSE calculations. BVSE maps with constant iso-surface energy of *E*_BVSE_(Li) over the global minimum indicate Na-ion diffusion pathways. BVSE calculations have been used to study a wide variety of ion conductors and show good agreement with experiments and calculations.

## Supplementary Material



## Notes

Additional experimental details, materials, and methods, including photographs of the experimental setup (PDF)
