# Supplementary material for: Synergistic Effect of Ternary Substitution in Na3V2(PO4)3 for High-Rate and Long-Life Anode-Free SIBs
Source: ACS Appl Mater Interfaces. 2025 Jul 21;17(32):45859–73. doi: 10.1021/acsami.5c08411 (PMC12356200; doi:10.1021/acsami.5c08411)
Supplement: Supplementary file 1 [file am5c08411_si_001.pdf]

Supplementary Information

Synergistic Effect of Ternary Substitution in $Na_3V_2(PO_4)_3$ for High-Rate and Long-Life anode-free SIBs

Ruoyu Chen[1], Dongdong Li[2], Xinyu Zhang[3,*], Shuoxiao Zhang[4], Xingyu Wang[4], Shilin Li[2], Il'ya A. Gural'skiy[5], Igor V. Zatovsky[6,*], Wei Han[2,*] and Denys Butenko[3,*]

[1] School of Mechanical and Intelligent Manufacturing, Fujian Chuanzheng Communications College, Fuzhou 350007, China.

[2] College of Physics, the State Key Laboratory of Inorganic Synthesis and Preparative Chemistry, International Center of Future Science, Jilin University, Changchun 130012, China. E-mail: whan@jlu.edu.cn

[3] Department of Physics and Institute of Major Scientific Facilities for New Materials, Southern University of Science and Technology, Shenzhen 518055, China. E-mail: debut98@ukr.net, zhangxy3@sustech.edu.cn

[4] Department of Physics, City University of Hong Kong, Hong Kong 999077, P. R. China.

[5] Department of Chemistry, Taras Shevchenko National University of Kyiv, Kyiv 01601, Ukraine.

[6] F.D. Ovcharenko Institute of Biocolloidal Chemistry, NAS Ukraine, Kyiv 03142, Ukraine. E-mail: zvigo@ukr.net

Features of the structural model for Rietveld refined XRD patterns

To perform Rietveld refined XRD patterns of the prepared samples, we used the results of X-ray structural studies of the single crystal $Na_3V_2(PO_4)_3$ (sp. gr. $R\overline{3}c$) as the starting structural model. This model assumes: one crystallographic site each for atoms V (Wyckoff position *12c*) and P (Wyckoff position *18e*); two crystallographic sites for sodium atoms (Na1 in Wyckoff positions *6b* and Na2 in Wyckoff positions *18e*) and two sites for oxygen atoms (O1 and O2 correspond to Wyckoff positions *36f*). However, substituting PO_4 by SO_4 groups in $Na_3V_2(PO_4)_3$ should lead to different values of P–O and S–O bond lengths for the local fragment of the structure. Based on this assumption, we modified the original structural data as follows:

For samples S2-NVP, S4-NVP, S6-NVP, KAS_2-NVP, KAS_4-NVP, and KAS_6-NVP, the crystallographic occupancies of sodium atoms, sulfur and phosphorous changes proportionally in line with contents calculated from the ratio of precursors. The fractional coordinates and the anisotropic displacement parameters of P1 and S1 were set to be the same. The P–O bond lengths were set at 1.53-1.54 Å, and the S–O bond lengths were set at 1.48 Å (P1–O1A and S1–O1B were modeled as co-directed, the same for P1–O2A and S1–O2B).

In addition, for the series of samples KAS_2-NVP, KAS_4-NVP, and KAS_6-NVP, it was assumed that aluminum atoms partially replace vanadium in V1 sites and potassium atoms partially replace sodium only in Na1 sites.

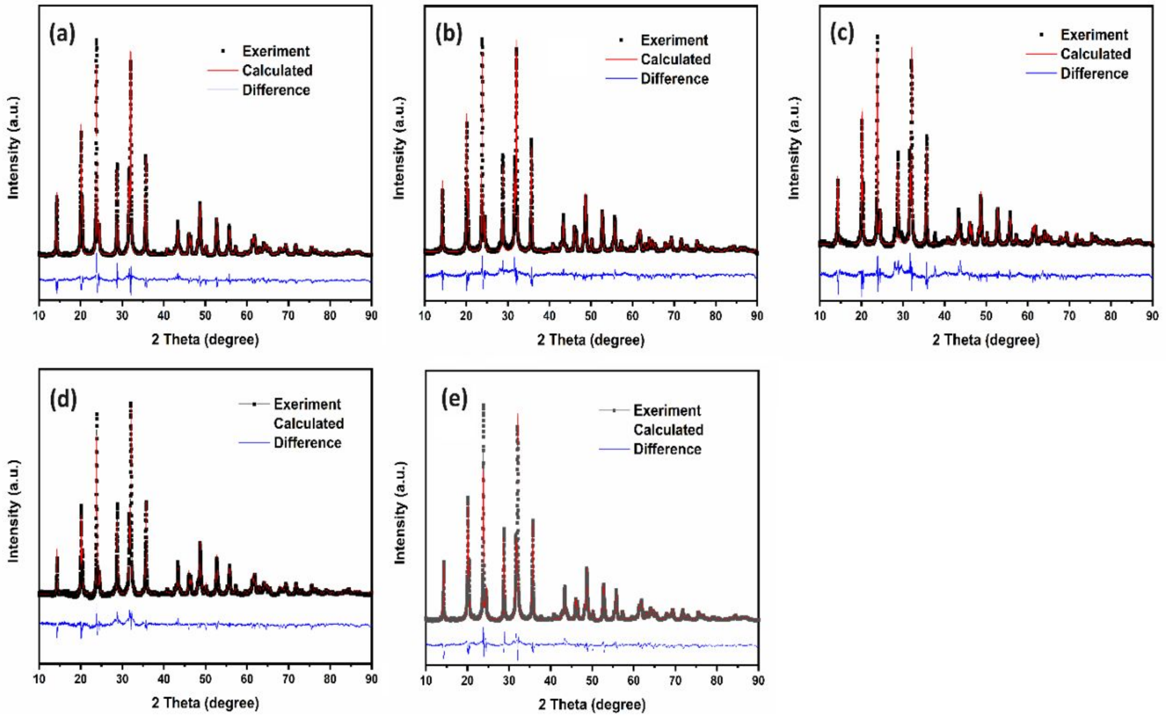

Figure S1. Rietveld refined XRD patters for (a) S2-NVP, (b) S4-NVP, (c) S6-NVP, (d) KAS_2-NVP, (e) KAS_6-NVP.

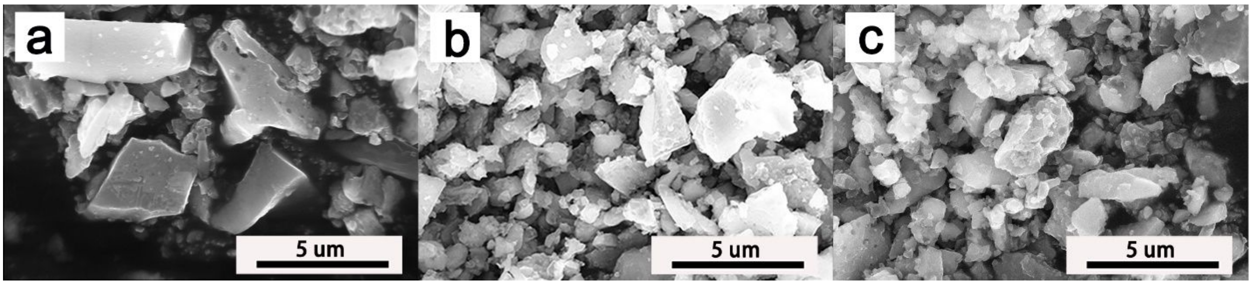

Figure S2. The SEM image of (a) NVP, (b) KAS_2-NVP and (c) KAS_6-NVP.

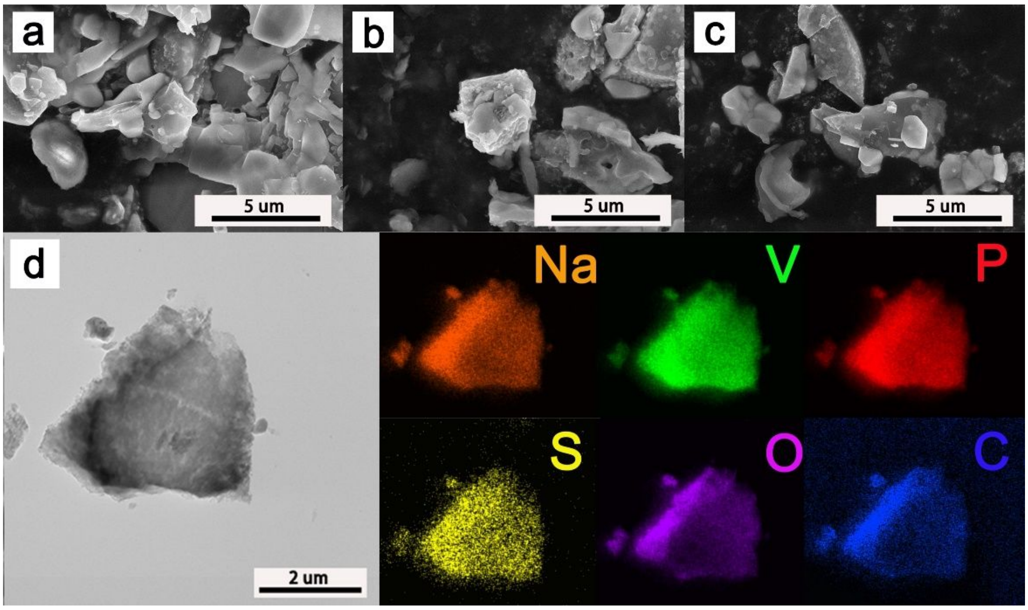

Figure S3. The SEM images of (a) S2-NVP, (b) S4-NVP and (c) S6-NVP; (d) the EDS element mapping of S4-NVP sample.

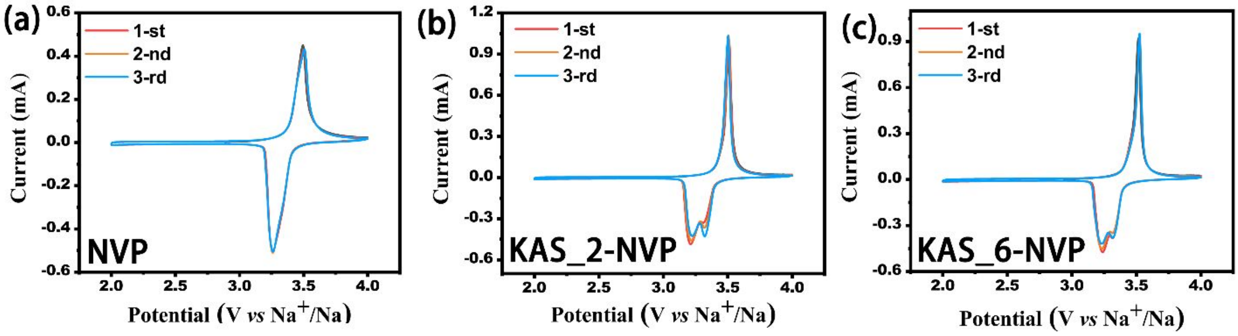

Figure S4. Cyclic voltammetry (CV) profiles at 0.2 mV s^{-1} for (a) NVP, (b) KAS_2-NVP and (c) KAS_6-NVP.

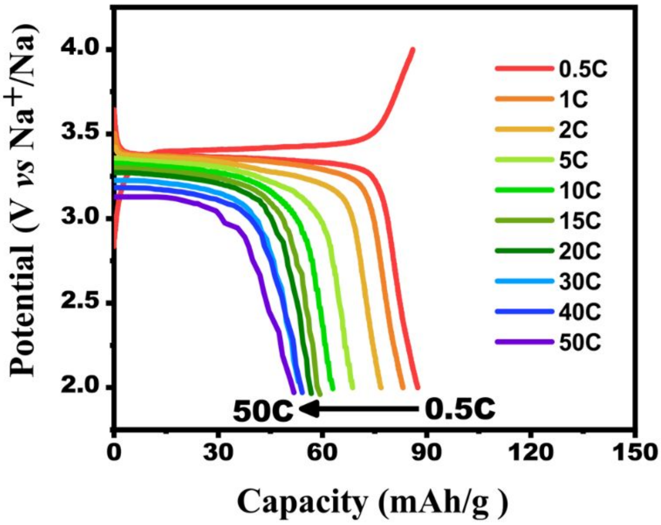

Figure S5. The charging/discharging profiles of NVP at different rates.

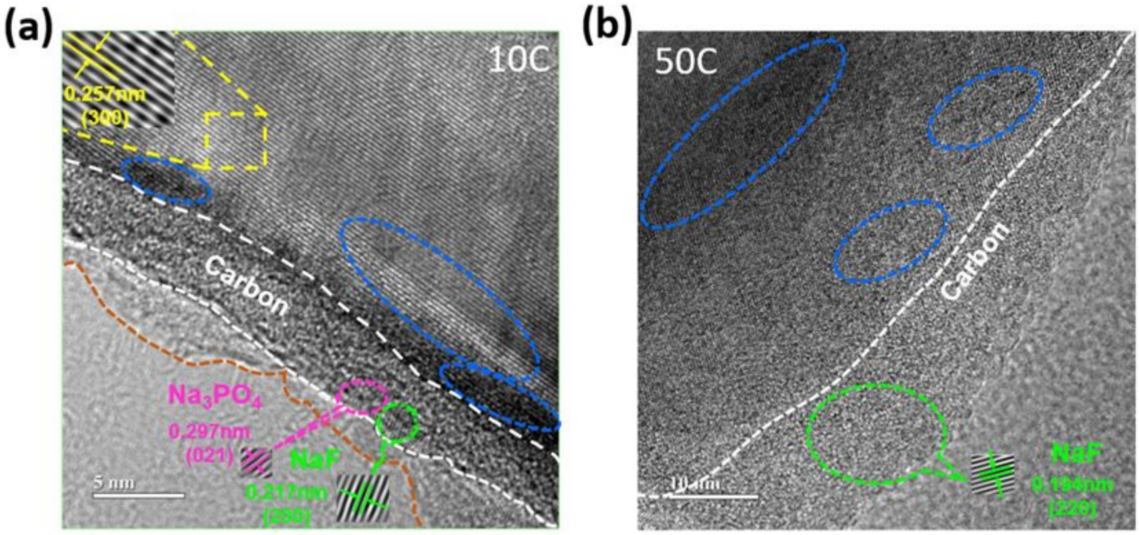

Figure S6. HRTEM image of the KAS_4-NVP electrodes before and after cycle performance of 500 cycles at (a) 10C and (b) 50C.

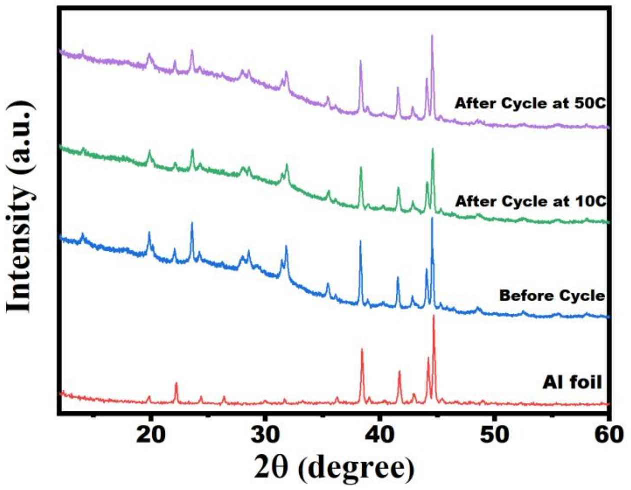

Figure S7. XRD patterns of the KAS_4-NVP electrodes before and after cycle performance of 500 cycles at 10C and 50C.

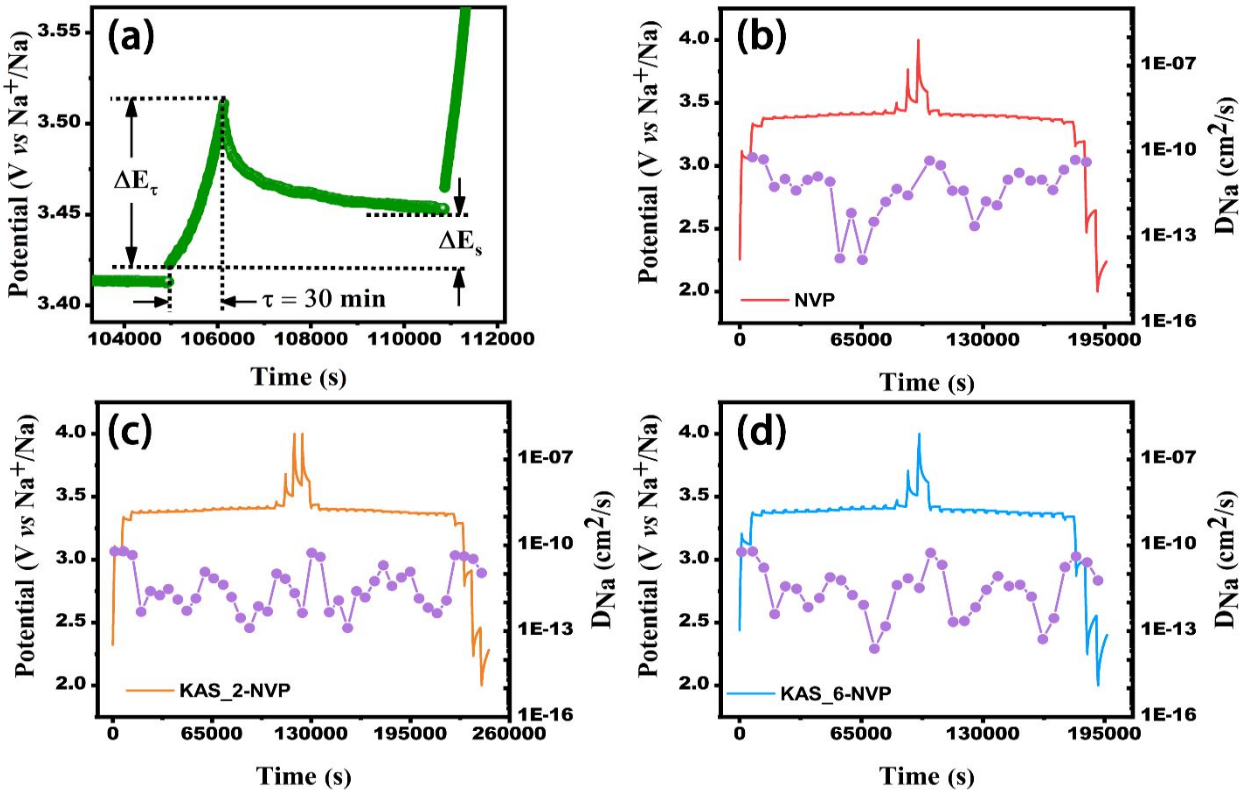

Figure S8. (a) Schematic illustration of selected steps in the Galvanostatic intermittent titration technique (GITT) profile during charging; GITT profiles and the corresponding calculated Na^+ diffusion coefficients (D_{Na^+}) of (b) NVP, (c) KAS_2-NVP and (d) KAS_6-NVP during charge/discharge processes at 0.1 C.

The diffusion coefficient (D_{Na^+}) can be calculated based on the equation (S1):

$$D_{Na^+} = \frac{4}{\pi\tau}\left(\frac{m_B V_M}{M_B A}\right)^2\left(\frac{\Delta E_S}{\Delta E_\tau}\right)^2 \quad \left(\tau \ll \frac{L^2}{D_{Na^+}}\right) \qquad (S1)$$

Where τ is the duration time of the current pulse, m_B is the mass of the active material, M_B is the molecular weight (483.84 g/mol), V_M is the molar volume (148.49 cm^3/mol), and A is the total contacting area of electrode with electrolyte (1.13 cm^2). ΔE_S is the difference between two consecutive stable voltages after relaxation, ΔE_τ is the transient voltage-change during a single titration step, and L is the thickness of electrode.

Sodium Diffusion Coefficients D_{Na}^+. The values of the sodium ion diffusion coefficient D_{Na}^+ can be approximated according to the Randles Sevick equation:

$$Ip = 2.69x10^{-5}n^{3/2}AD_{Na}^{1/2}C_{Na}v^{1/2} \quad \text{(S2)}$$

Where Ip is the peak current of the reduction peak or the oxidation peak, n is the number of transferred electrons, A is the effective contact area between the electrode and the electrolyte (here the usual value is 1.13 cm^2), C_{Na} is the concentration of sodium ions in the electrode (2.69×10^{-2} mol/cm^3). D_{Na} is the diffusion coefficient of sodium ions, and v is the scan rate.

Pseudocapacitive Contribution Calculation. The pseudocapacitive contributions to Na storage in KAS_4-NVP composite were calculated by following equations:

$$i=k_1v+k_2v^{1/2} \quad \text{(S3)}$$

Where k_1v and $k_2v^{1/2}$ represent the capacitive and diffusion-limited currents, respectively.

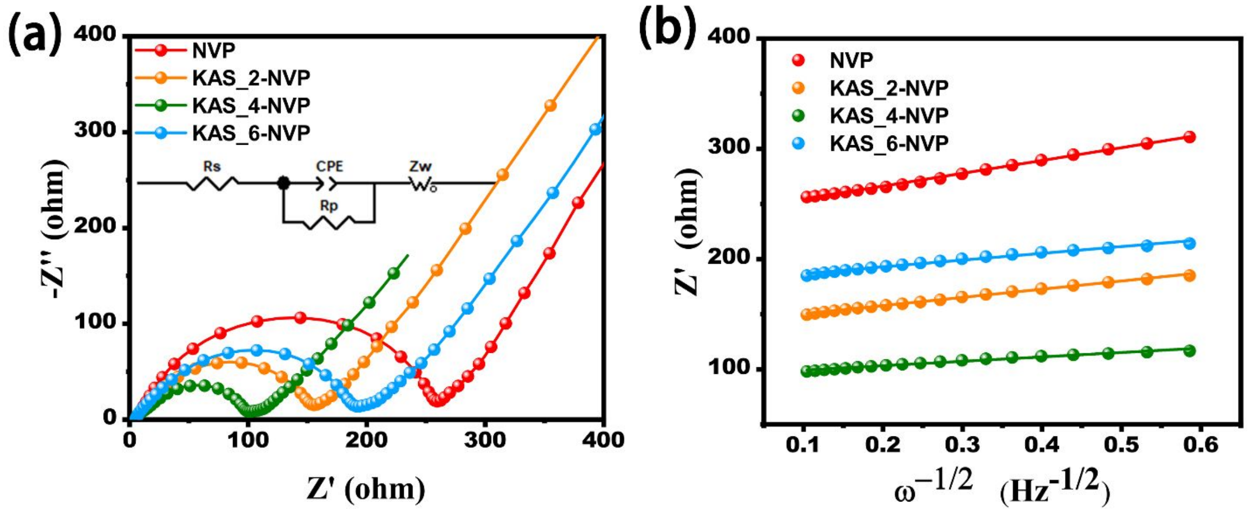

Figure S9. (a) Nyquist plots of KAS-NVP series samples after CV at 0.2 mV s^{-1}, inset, the equivalent electrical circuit for the EIS; (b) Z' and $\omega^{-1/2}$ relationship.

Nyquist graphs for all prepared electrodes charged to 4 V after CV at 0.2 mV s^{-1} are shown in **Figure S9**a and **S11**a. The EIS curves are a semicircle in the high frequency region, which corresponds to the charge transfer resistance, and a straight line in the low frequency region, which corresponds to the diffusion resistance of sodium ions in the electrode. The equivalent circuit used to fit the EIS (insert in **Figure S9**a and **S11**a) is convenient for further analysis of the impedance spectrum, where R_s represents the resistance of the electrolyte and R_p refers to the charge transfer resistance. CPE corresponds to the double layer capacitance and capacity of the surface layer, and Warburg impedance (Z_w) is related to the solid-state diffusion of ions in the active material and corresponds to the slopping line in the low-frequency region.

Calculations of the diffusion coefficient (D_{Na+}) of sodium ions. To estimate the diffusion coefficient of sodium ions (D_{Na+}), the low-frequency region of the EIS spectra is used. The diffusion kinetics can be determined according to the following equations:

$$\mathrm{D} = \frac{R^2T^2}{2A^2n^4F^4C^2\sigma^2} \qquad \text{(S6)},$$

$$Z' = R_s + R_p + \sigma\omega^{-1/2} \qquad \text{(S7)},$$

where R is the gas constant (8.314 J mol^{-1} K^{-1}), T stands for the absolute temperature (K), A is the contacting area of electrode with electrolyte (cm^2), n is the number of transferred electrons, F is the Faraday constant (96500 C mol^{-1}), C is the concentration of Na ions in the cathode electrode (3.47 x 10^{-3} mol cm^{-3}), given the chemical composition of the active material, σ is the Warburg factor which can be calculated from Z'. The D_{Na}^{+} can be calculated from equation (S5), σ is related to Z' through equation (S6), and its value can be determined from the slope of the line between Z' and $\omega^{1/2}$ (**Figure S9**b and **S11**b).

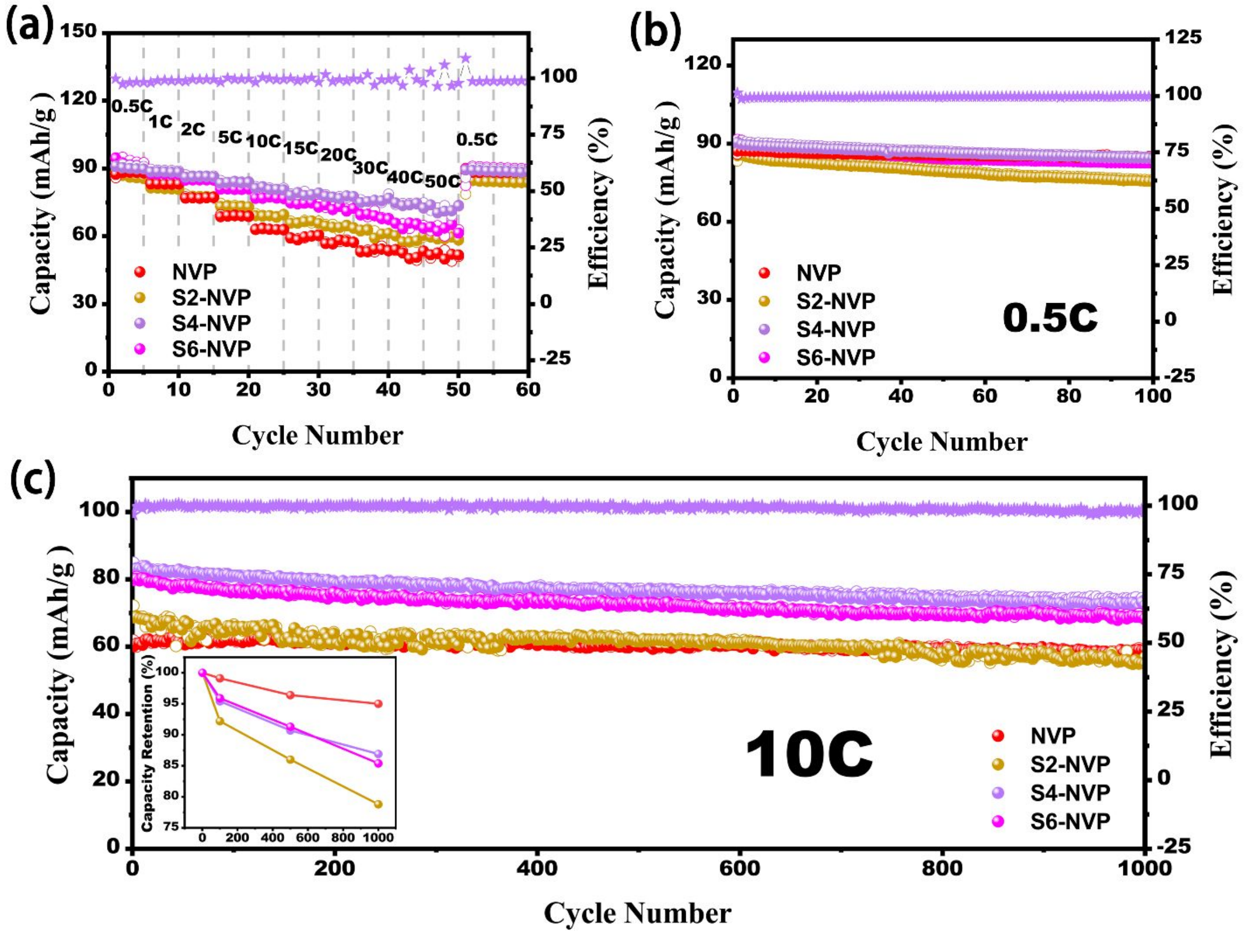

Figure S10. Electrochemical performance of $Na_{3-x}V_2(PO_4)_{3-x}(SO_4)_x/C$ (x = 0.02, 0.04, 0.06) cathodes within the voltage window of 2.0-4.0 V versus Na^+/Na: (a) Rate capability at different rates; (b) Cycling stability at 0.5 C and (c) Long-term cycle performance at a high rate of 10 C (inset: capacity retention, %).

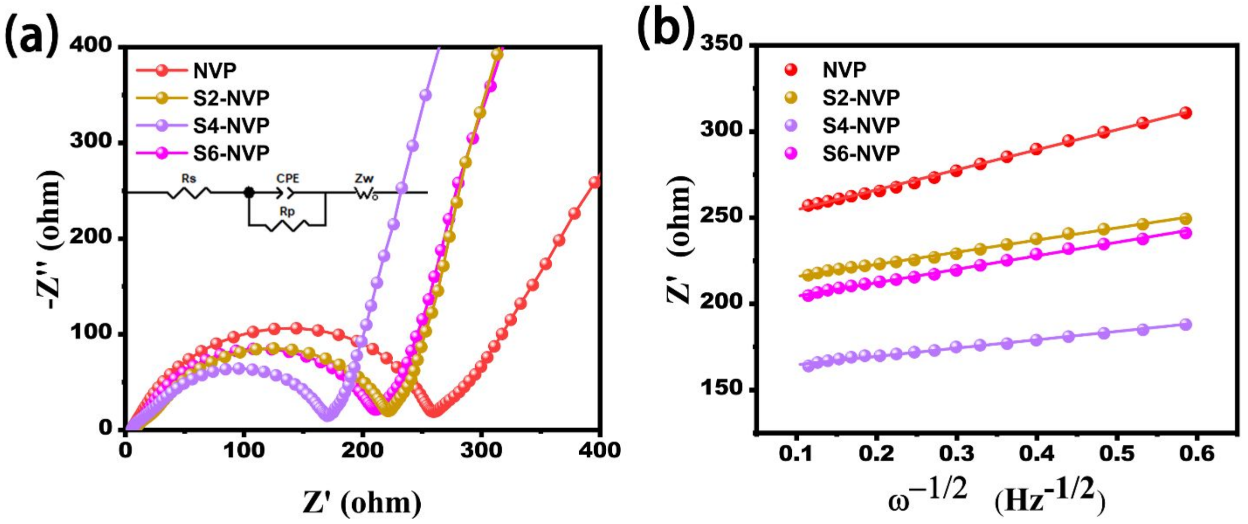

FigureS11. (a) Nyquist plots of S-NVP series samples after CV at 0.2 mV s^{-1}, inset, the equivalent electrical circuit for the EIS; (b) Z’ and $\omega^{-1/2}$ relationship.

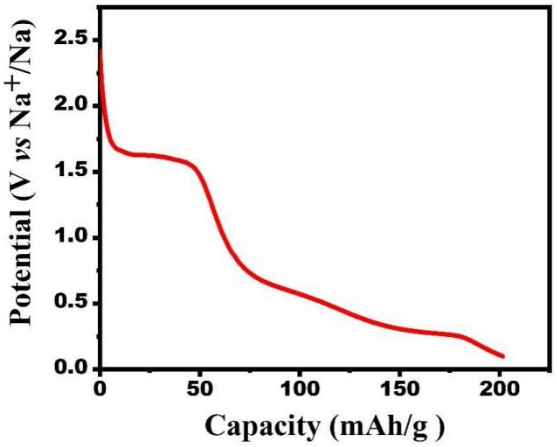

Figure. S12 The initial discharge curve of KAS_4-NVP electrode at 0.5 C.

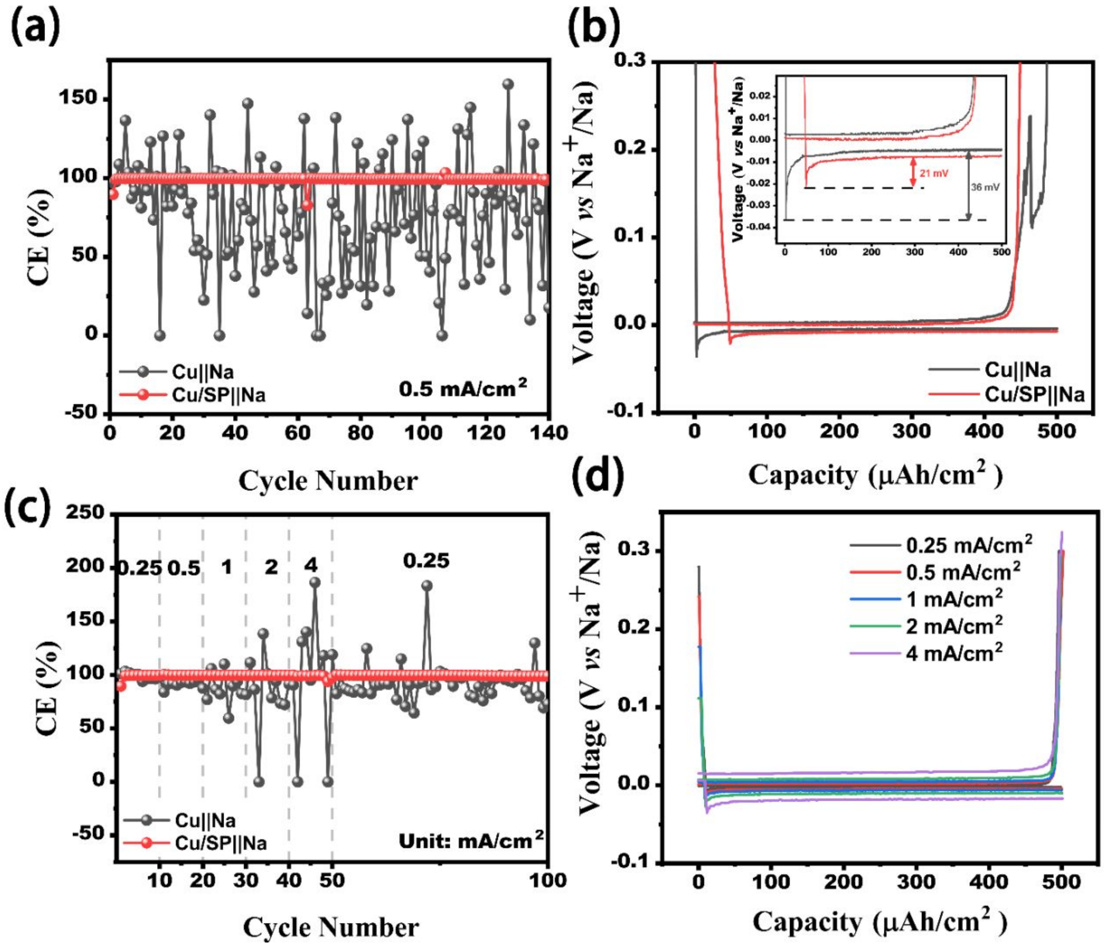

Figure S13. Sodium plating/stripping behaviors on Cu/SP and Cu current collectors. (a) Coulombic efficiencies (CE) and (b) voltage profiles of Na||Cu/SP and Na||Cu cells at 0.5 mA cm^{-2} with a Na deposition capacity of 0.5 mAh cm^{-2}; (c) CE of Na||Cu/SP and Na||Cu cells at various areal currents with a deposition capacity of 0.5 mAh cm^{-2}, and (d) corresponding voltage profiles of Na||Cu/SP cells.

To investigate the effect of Super P layer on Na deposition/stripping on the surface of Cu foil during charging and discharging, the electrochemical performance test on Na||Cu/SP and Na||Cu symmetric batteries at different current densities is preformed (**Figure 13**). The Coulombic efficiencies (CE) and voltage profiles of Na||Cu/SP and Na||Cu cells at 0.5 mA cm^{-2} with a Na deposition capacity of 0.5 mAh cm^{-2} are shown in **Figure S13**a and b. It can be clearly seen that the cycle stability of Cu/SP foil is significantly superior to that of bare copper at 0.5 mA cm^{-2}, and its CE remains around 100% after 140 cycles (**Figure S13**a). Moreover, the nucleation over potential of Cu/SP foil (21 mV) is significantly lower than that of Cu foil (36 mV), which is more conducive to the Na deposition/stripping on the surface of the collector (insert in **Figure S13**b). In addition, Cu/SP foil also exhibits excellent rate performance at different rates (**Figure S13**c and d).

Calculation of specific energy/power density

The load mass of the SP layer on Cu foil is approximately 0.1-0.2 mg cm^{-2}, which is much lower than the mass of active materials in the KAS_4-NVP cathode (4-7 mg). Therefore, the energy density and power density of KAS_4-NVP||Cu/SP SFA-SMBs was calculated by the following equation:

$$E=\frac{Q*V}{M_{KAS\_4-NVP}}, \qquad (S8)$$

$$P=\frac{E}{t}. \qquad (S9)$$

Herein, E represents the energy density (Wh kg^{-1}). Q (mAh) and V are the discharge capacity and average discharge voltage of the cell. $M_{KAS\_4\text{-}NVP}$ is the mass loading of the KAS_4-NVP in the cathode. P represents the power density (W kg^{-1}), and t is the time for full discharge. As mentioned above, the energy density (E) and power density (P) of the cells are calculated by considering the total mass of active materials in electrodes (no mass at the anode side for SFA-SMBs).

Table S1. Rietveld refinement results for S2-NVP, S4-NVP, S6-NVP and KAS_2-NVP, KAS_4-NVP, KAS_6-NVP series samples.

Sample and composition	Cell parameters and Rietveld refinement result
**S2-NVP**	**a = b = 8.71421(25) Å, c = 21.8427(4) Å, V = 1436.46(5) Å^3**
**$Na_{2.98}V_2(PO_4)_{2.98}(SO_4)_{0.02}$**	**R_p = 5.37%, R_{wp} = 7.15%, R_b = 6.88%, R_{wb} = 7.50%**
**S4-NVP**	**a = b = 8.7297(4) Å, c = 21.8549(7) Å, V = 1442.37(10) Å^3**
**$Na_{2.96}V_2(PO_4)_{2.96}(SO_4)_{0.04}$**	**R_p = 4.78%, R_{wp} = 6.15%, R_b = 5.58%, R_{wb} = 5.98%**
**S6-NVP**	**a = b = 8.7323(5) Å, c = 21.8643(9) Å, V = 1443.86(14) Å^3**
**$Na_{2.94}V_2(PO_4)_{2.94}(SO_4)_{0.06}$**	**R_p = 4.89%, R_{wp} = 6.76%, R_b = 6.22%, R_{wb} = 7.03%**
**KAS_2-NVP**	**a = b = 8.71859(28) Å, c = 21.8709(4) Å, V = 1439.76(5) Å^3**
**$Na_{2.97}K_{0.01}V_{1.99}Al_{0.01}(PO_4)_{2.98}(SO_4)_{0.02}$**	**R_p = 3.67%, R_{wp} = 4.61%, R_b = 5.61%, R_{wb} = 6.26%**
**KAS_4-NVP**	**a = b = 8.7165(4) Å, c = 21.8732(5) Å, V = 1439.23(7) Å^3**
**$Na_{2.94}K_{0.02}V_{1.98}Al_{0.02}(PO_4)_{2.96}(SO_4)_{0.04}$**	**R_p = 3.60%, R_{wp} = 4.48%, R_b = 4.31%, R_{wb} = 4.85%**
**KAS_6-NVP**	**a = b = 8.7211(3) Å, c = 21.8552(5) Å, V = 1439.56(6) Å^3**
**$Na_{2.91}K_{0.03}V_{1.97}Al_{0.03}(PO_4)_{2.94}(SO_4)_{0.06}$**	**R_p = 5.18%, R_{wp} = 7.35%, R_b = 6.62%, R_{wb} = 8.16%**

Table S2. Structural parameters of S2-NVP sample deduced from Rietveld refinement.

Atom	Wyck.	x	y	z	B	Occ.
V1	12c	0.66667	1.33333	-0.01950	0.0069	1.0000
P1	18e	0.62394	0.95727	-0.08333	0.0087	0.9933
S1	18e	0.62394	0.95727	-0.08333	0.0087	0.0067
Na1	6b	0.66667	1.33333	-0.16667	0.1491	0.8036
Na2	18e	0.33333	1.03275	-0.08333	0.0522	0.7255
O1A	36f	0.64431	1.14206	-0.07761	0.0164	0.9933
O1B	36f	0.64291	1.13527	-0.07833	0.0164	0.0067
O2A	36f	0.54042	0.84474	-0.02640	0.0259	0.9933
O2B	36f	0.54284	0.84840	-0.02804	0.0259	0.0067

Table S3. Structural parameters of S4-NVP sample deduced from Rietveld refinement.

Atom	Wyck.	x	y	z	B	Occ.
V1	12c	0.66667	1.33333	-0.01950	0.0069	1.0000
P1	18e	0.62394	0.95727	-0.08333	0.0087	0.9833
S1	18e	0.62394	0.95727	-0.08333	0.0087	0.0133
Na1	6b	0.66667	1.33333	-0.16667	0.1491	0.7982
Na2	18e	0.33333	1.03275	-0.08333	0.0522	0.7206
O1A	36f	0.64431	1.14206	-0.07761	0.0164	0.9867
O1B	36f	0.64291	1.13527	-0.07833	0.0164	0.0133
O2A	36f	0.54042	0.84474	-0.02640	0.0259	0.9867
O2B	36f	0.54284	0.84840	-0.02804	0.0259	0.0133

Table S4. Structural parameters of S6-NVP sample deduced from Rietveld refinement.

Atom	Wyck.	x	y	z	B	Occ.
V1	12c	0.66667	1.33333	-0.01950	0.0069	1.0000
P1	18e	0.62394	0.95727	-0.08333	0.0087	0.9800
S1	18e	0.62394	0.95727	-0.08333	0.0087	0.0200
Na1	6b	0.66667	1.33333	-0.16667	0.1491	0.7918
Na2	18e	0.33333	1.03275	-0.08333	0.0522	0.7154
O1A	36f	0.64431	1.14206	-0.07761	0.0164	0.9800
O1B	36f	0.64291	1.13527	-0.07833	0.0164	0.0200
O2A	36f	0.54042	0.84474	-0.02640	0.0259	0.9800
O2B	36f	0.54284	0.84840	-0.02804	0.0259	0.0200

Table S5. Structural parameters of KAS_2-NVP sample deduced from Rietveld refinement.

Atom	Wyck.	x	y	z	B	Occ.
V1	12c	0.66667	1.33333	-0.01950	0.0069	0.9950
Al1	12c	0.66667	1.33333	-0.01950	0.0069	0.0050
P1	18e	0.62394	0.95727	-0.08333	0.0087	0.9933
S1	18e	0.62394	0.95727	-0.08333	0.0087	0.0067
Na1	6b	0.66667	1.33333	-0.16667	0.1491	0.7800
K1	6b	0.66667	1.33333	-0.16667	0.1491	0.0100
Na2	18e	0.33333	1.03275	-0.08333	0.0522	0.7300
O1A	36f	0.64431	1.14206	-0.07761	0.0164	0.9933
O1B	36f	0.64291	1.13527	-0.07833	0.0164	0.0067
O2A	36f	0.54042	0.84474	-0.02640	0.0259	0.9933
O2B	36f	0.54284	0.84840	-0.02804	0.0259	0.0067

Table S6. Structural parameters of KAS_4-NVP sample deduced from Rietveld refinement.

Atom	Wyck.	x	y	z	B	Occ.
V1	12c	0.66667	1.33333	-0.01950	0.0069	0.9900
Al1	12c	0.66667	1.33333	-0.01950	0.0069	0.0100
P1	18e	0.62394	0.95727	-0.08333	0.0087	0.9867
S1	18e	0.62394	0.95727	-0.08333	0.0087	0.0133
Na1	6b	0.66667	1.33333	-0.16667	0.1491	0.7500
K1	6b	0.66667	1.33333	-0.16667	0.1491	0.0200
Na2	18e	0.33333	1.03275	-0.08333	0.0522	0.7300
O1A	36f	0.64431	1.14206	-0.07761	0.0164	0.9867
O1B	36f	0.64291	1.13527	-0.07833	0.0164	0.0133
O2A	36f	0.54042	0.84474	-0.02640	0.0259	0.9867
O2B	36f	0.54284	0.84840	-0.02804	0.0259	0.0133

Table S7. Structural parameters of KAS_6-NVP sample deduced from Rietveld refinement.

Atom	Wyck.	x	y	z	B	Occ.
V1	12c	0.66667	1.33333	-0.01950	0.0069	0.9850
Al1	12c	0.66667	1.33333	-0.01950	0.0069	0.0150
P1	18e	0.62394	0.95727	-0.08333	0.0087	0.9800
S1	18e	0.62394	0.95727	-0.08333	0.0087	0.0200
Na1	6b	0.66667	1.33333	-0.16667	0.1491	0.7200
K1	6b	0.66667	1.33333	-0.16667	0.1491	0.0300
Na2	18e	0.33333	1.03275	-0.08333	0.0522	0.7300
O1A	36f	0.64431	1.14206	-0.07761	0.0164	0.9867
O1B	36f	0.64291	1.13527	-0.07833	0.0164	0.0133
O2A	36f	0.54042	0.84474	-0.02640	0.0259	0.9800
O2B	36f	0.54284	0.84840	-0.02804	0.0259	0.0200

Table S8. The actual and theoretical mass content values (ICP $_{actual}$ and ICP $_{theoretical}$, wt%) of different elements for KAS_4-NVP sample.

	Na	V	P	K	Al	S
ICP $_{actual}$, wt%	13.383	19.393	18.642	0.438	0.194	0.401
ICP $_{theoretical}$, wt%	14.931	21.906	20.045	0.343	0.236	0.562

Table S9. The cycling performance of $Na_{3-1.5x}K_{0.5x}V_{2-0.5x}Al_{0.5x}(PO_4)_{3-x}(SO_4)_x/C$ and $Na_{3-x}V_2(PO_4)_{3-x}(SO_4)_x/C$ (x = 0, 0.02, 0.04, 0.06) cathodes at 0.5 C in a potential of 2.0-4.0 V, mAh g^{-1} (capacity retention is indicated in %).

Sample	Number of cycles	
	1	100
NVP	87.2	84.8 (97.2%)
KAS_2-NVP	105.9	100.5 (94.9%)
KAS_4-NVP	115.3	105.3 (91.3%)
KAS_6-NVP	92.1	85.0 (92.2%)
S2-NVP	85.4	75.5 (88.4%)
S4-NVP	90.6	84.4 (93.2%)
S6-NVP	90.4	82.6 (91.3%)

Table S10. The long-term cycle performance of $Na_{3-1.5x}K_{0.5x}V_{2-0.5x}Al_{0.5x}(PO_4)_{3-x}(SO_4)_x$/C and $Na_{3-x}V_2(PO_4)_{3-x}(SO_4)_x$/C (x = 0, 0.02, 0.04, 0.06) cathodes at 10 C in a potential of 2.0-4.0 V, mAh g^{-1} (capacity retention is indicated in %).

Sample	Number of cycles			
	1	100	500	1000
**NVP**	62.0	61.5 (99.1%)	59.8 (96.4%)	58.9 (95.0%)
**KAS_2-NVP**	83.3	81.1 (97.4%)	78.5 (94.2%)	75.2 (90.3%)
**KAS_4-NVP**	92.9	90.3 (97.2%)	86.9 (93.5%)	83.6 (90.0%)
**KAS_6-NVP**	70.5	68.1 (96.6%)	65.3 (92.6%)	62.7 (89.0%)
**S2-NVP**	72.2	66.6 (92.2%)	62.1 (86.0%)	56.9 (78.8%)
**S4-NVP**	85.0	81.1 (95.4%)	77.1 (90.7%)	73.9 (86.9%)
**S6-NVP**	80.1	76.8 (95.9%)	73.1 (91.3%)	68.4 (85.4%)

Table S11. A comparison of the electrochemical performance of prepared KAS-NVP and S-NVP sampes with the similar single ion or multi ions doped NVP electrodes.

Composition	Specific Capacity (mAh/g)	Cycle Number	C-rate	Capacity Retention (%)	Ref.
**$Na_3.V_2(PO_4)_3$**	**87.2**	**100**	**0.5C**	**97.2**	**This work**
	**62.0**	**1000**	**10C**	**95.0**	
K^+, Al^{3+} and SO_4^{2-}-doped NVP					
**$Na_{2.97}K_{0.01}V_{1.99}Al_{0.01}(PO_4)_{2.98}(SO_4)_{0.02}$**	**105.9**	**100**	**0.5C**	**94.9**	**This work**
	**83.3**	**1000**	**10C**	**90.3**	
**$Na_{2.94}K_{0.02}V_{1.98}Al_{0.02}(PO_4)_{2.96}(SO_4)_{0.04}$**	**115.3**	**100**	**0.5C**	**91.3**	**This work**
	**92.9**	**1000**	**10C**	**90.0**	
	**79.1**	**4000**	**50C**	**72.8**	
	**79.1**	**10000**	**50C**	**55.8**	
**$Na_{2.91}K_{0.03}V_{1.97}Al_{0.03}(PO_4)_{2.94}(SO_4)_{0.06}$**	**92.1**	**100**	**0.5C**	**88.9**	**This work**
	**70.5**	**1000**	**10C**	**89.0**	
SO_4^{2-}-doped NVP					
**$Na_{2.98}V_2(PO_4)_{2.98}(SO_4)_{0.02}$**	**85.4**	**100**	**0.5C**	**88.4**	**This work**
	**72.2**	**1000**	**10**	**78.8**	
**$Na_{2.96}V_2(PO_4)_{2.96}(SO_4)_{0.04}$**	**90.6**	**100**	**0.5C**	**93.2**	**This work**
	**85.0**	**1000**	**10C**	**86.9**	
**$Na_{2.94}V_2(PO_4)_{2.94}(SO_4)_{0.06}$**	**90.4**	**100**	**0.5C**	**91.3**	**This work**
	**80.1**	**1000**	**10C**	**85.4**	
Al^{3+}-doped NVP					
$Na_3V_{1.98}Al_{0.02}(PO_4)_3$	95.8	50	0.6C	99.2	[1]
$Na_3V_{1.8}Al_{0.2}(PO_4)_3$	117.1	100	1C	95	[2]
K^+-doped NVP					
$Na_{2.88}K_{0.12}V_2(PO_4)_3$	107.3	200	0.2C	~83.9	[3]
	~100	200	1C	~83	
K^+, La^{3+} and SiO_4^{4-}-doped NVP (This could be explained by K^+ ions enlarging the height of the crystal bulk for superfluous sodium ions to insert into the R3c space group,)					

$Na_{3.03}V_{1.93}La_{007}(PO_4)_{2.9}(SiO_4)_{0.1}$	104.8	200	1C	90.6	[4]
	109.3	1500	10C	83.2	
	84.8	1300	50C	69.8	
K^+ and Ca^{2+}-doped NVP					
$Na_{2.79}K_{0.07}Ca_{0.07}V_2(PO_4)_3$	99.4	50	0.1C	91	[5]
	88.6	150	1C	83	
K^+ and Co^{2+}-doped NVP					
$Na_{2.95}K_{0.1}V_{1.95}Co_{0.05}(PO_4)_3$	100.9	500	1C	70.4	[6]
	98.8	400	10C	75.9	
K^+ and Zr^{4+}-doped NVP					
$Na_{2.95}K_{0.04}V_{1.93}Zr_{0.0525}(PO_4)_3$	100.3	400	2C	92.02	[7]
Zr^{4+} and SiO_4^{4-}-doped NVP					
$Na_3V_{1.97}Zr_{0.03}(PO_4)_{2.97}(SiO_4)_{0.03}$	107.7	500	1C	90.3	[8]
	103.8	500	6C	93.9	
	97.3	2000	12C	70.2	
SiO_4^{4-}-doped NVP					
$Na_{3.05}V_2(PO_4)_{2.95}(SiO_4)_{0.05}$	~100	250	0.5C	82	[9]
Co^{2+}-doped NVP					
$Na_{3.01}V_{1.99}Co_{0.01}(PO_4)_3$	116	100	0.5C	92.2	[10]
	106	1000	10C	83.3	
Cu^{2+}-doped NVP					
$Na_{3.03}V_{1.97}Cu_{0.03}(PO_4)_3$	114	100	0.5C	87.7	[10]
	92	1000	10C	78.8	
Ni^{2+}-doped NVP					
$Na_{3.03}V_{1.97}Ni_{0.03}(PO_4)_3$	113.4	100	0.5C	90.5	[11]
	107.1	100	1C	95.5	
$Na_3V_{1.97}Ni_{0.03}(PO_4)_3$	98.1	50	5C	93.5	[12]
$Na_{3.1}V_{1.9}Ni_{0.1}(PO_4)_3$	109	100	1C	94.7	[13]
Mn^{2+}-doped NVP					
$Na_3V_{1.875}Mn_{0.025}(PO_4)_3$	89.2	100	10C	97.2	[14]
	86.7	100	15C	91.6	
$Na_3V_{1.9}Mn_{0.1}(PO_4)_3$	113	100	1C	95	[15]
	78	50	10C	90	
$Na_3V_{1.8}Mn_{0.2}(PO_4)_3$	77.8	10000	30C	82	[16]
Mg^{2+}-doped NVP					
$Na_3V_{1.95}Mg_{0.05}(PO_4)_3$	86.2	50	20C	81	[17]
$Na_3V_{1.95}Mg_{0.05}(PO_4)_3$	102	108	1C	90.2	[18]
	96.7	180	10C	88.9	
$Na_3V_{1.93}Mg_{0.07}(PO_4)_3$	95	1000	10C	84.6	[19]
$Na_{3.1}V_{1.9}Mg_{0.1}(PO_4)_3$	113	100	1C	94	[13]

Table S12. The long-term cycle performance of KAS_4-NVP cathode at 50 C in a potential of 2.0-4.0 V, mAh g^{-1} (capacity retention is indicated in %).

	Number of cycles					
	1	1000	2000	3000	4000	5000
Capacity	84.4	68.1	64.0	63.8	60.8	54.0
Capacity Retention	100%	80.7%	75.8%	75.6%	72.0%	64.0%
	Number of cycles					
	6000	7000	8000	9000	10000	
Capacity	51.4	50.9	49.5	48.8	48.2	
Capacity Retention	60.9%	60.3%	58.6%	57.8%	57.1%	

Table S13. Kinetic Parameters and Sodium Diffusion Coefficients D_{Na}^{+} of KAS-NVP and S-NVP Series Samples were obtained from Equivalent Circuit Fitting.

Sample	R_s/Ω	R_p/Ω	D/(cm^2 s^{-1})
NVP	5.04	258.1	6.58×10^{-13}
KAS_2-NVP	4.83	157.3	1.62×10^{-12}
KAS_4-NVP	4.48	100.2	5.56×10^{-12}
KAS_6-NVP	4.89	190.8	2.36×10^{-12}
S2-NVP	8.27	197.7	1.81×10^{-12}
S4-NVP	4.92	152.5	3.86×10^{-12}
S6-NVP	6.17	210.5	1.46×10^{-12}

References

[1] Y. Chen, Y. Xu, X. Sun, C. Wang, Effect of Al substitution on the enhanced electrochemical performance and strong structure stability of Na3V2(PO4)3/C composite cathode for sodium-ion batteries, Journal of Power Sources 375 (2018) 82-92.

[2] L. Zhao, H. Zhao, Z. Du, N. Chen, X. Chang, Z. Zhang, F. Gao, A. Trenczek-Zajac, K. Świerczek, Computational and experimental understanding of Al-doped Na3V2-xAlx(PO4)3 cathode material for sodium ion batteries: Electronic structure, ion dynamics and electrochemical properties, Electrochimica Acta 282 (2018) 510-519.

[3] S.-J. Lim, D.-W. Han, D.-H. Nam, K.-S. Hong, J.-Y. Eom, W.-H. Ryu, H.-S. Kwon, Structural enhancement of Na3V2(PO4)3/C composite cathode materials by pillar ion doping for high power and long cycle life sodium-ion batteries, Journal of Materials Chemistry A 2(46) (2014) 19623-19632.

[4] S. Sun, Y. Chen, Q. Bai, Q. Huang, C. Liu, S. He, Y. Yang, Y. Wang, L. Guo, Unraveling the modified regulation of ternary substitution on Na3V2(PO4)3 for sodium ion batteries, Journal of Materials Chemistry A 10(21) (2022) 11340-11353.

[5] Q. Zhu, H. Cheng, X. Zhang, L. He, L. Hu, J. Yang, Q. Chen, Z. Lu, Improvement in electrochemical performance of Na3V2(PO4)3/C cathode material for sodium-ion batteries by K-Ca co-doping, Electrochimica Acta 281 (2018) 208-217.

[6] Z. Tian, Y. Chen, J. Cheng, S. Sun, C. Wang, Z. He, X. Shi, Y. Wang, L. Guo, Boosting the rate capability and working lifespan of K/Co co-doped Na3V2(PO4)3/C for sodium ion batteries, Ceramics International 47(15) (2021) 22025-22034.
[7] J. Li, J. Cheng, Y. Chen, C. Wang, L. Guo, Effect of K/Zr co-doping on the elevated electrochemical performance of Na3V2(PO4)3/C cathode material for sodium ion batteries, Ionics 27(1) (2021) 181-190.
[8] J. Cheng, Y. Chen, Y. Wang, C. Wang, Z. He, D. Li, L. Guo, Insights into the enhanced sodium storage property and kinetics based on the Zr/Si codoped Na3V2(PO4)3/C cathode with superior rate capability and long lifespan, Journal of Power Sources 474 (2020) 228632.
[9] M.J. Aragón, P. Lavela, G.F. Ortiz, R. Alcántara, J.L. Tirado, On the Effect of Silicon Substitution in Na3V2(PO4)3 on the Electrochemical Behavior as Cathode for Sodium-Ion Batteries, ChemElectroChem 5(2) (2018) 367-374.
[10] R. Chen, D.S. Butenko, S. Li, D. Li, X. Zhang, J. Cao, I.V. Ogorodnyk, N.I. Klyui, W. Han, I.V. Zatovsky, Effects of low doping on the improvement of cathode materials Na3+xV2−xMx(PO4)3 (M = Co2+, Cu2+; x = 0.01–0.05) for SIBs, Journal of Materials Chemistry A 9(32) (2021) 17380-17389.
[11] H. Li, Y. Bai, F. Wu, Q. Ni, C. Wu, Na-Rich Na3+xV2–xNix(PO4)3/C for Sodium Ion Batteries: Controlling the Doping Site and Improving the Electrochemical Performances, ACS Applied Materials & Interfaces 8(41) (2016) 27779-27787.
[12] B. Zhang, H. Chen, H. Tong, X. Wang, J. Zheng, W. Yu, J. Zhang, J. Li, W. Zhang, Synthesis and electrochemical performance of Ni doped Na3V2(PO4)3/C cathode materials for sodium ion batteries, Journal of Alloys and Compounds 728 (2017) 976-983.
[13] S. Bag, H. Murarka, C. Zhou, A. Bhattacharya, D. Jokhakar, V.G. Pol, V. Thangadurai, Understanding the Na-Ion Storage Mechanism in Na3+xV2–xMx(PO4)3 (M = Ni2+, Co2+, Mg2+; x = 0.1–0.5) Cathodes, ACS Applied Energy Materials 3(9) (2020) 8475-8486.
[14] W. Shen, H. Li, Z. Guo, Z. Li, Q. Xu, H. Liu, Y. Wang, Improvement on the high-rate performance of Mn-doped Na3V2(PO4)3/C as a cathode material for sodium ion batteries, RSC Advances 6(75) (2016) 71581-71588.
[15] J.-S. Park, J. Kim, J.H. Jo, S.-T. Myung, Role of the Mn substituent in Na3V2(PO4)3 for high-rate sodium storage, Journal of Materials Chemistry A 6(34) (2018) 16627-16637.
[16] J. Fang, S. Wang, X. Yao, X. Hu, Y. Wang, H. Wang, Ration design of porous Mn-doped Na3V2(PO4)3 cathode for high rate and super stable sodium-ion batteries, Electrochimica Acta 295 (2019) 262-269.
[17] H. Li, X. Yu, Y. Bai, F. Wu, C. Wu, L.-Y. Liu, X.-Q. Yang, Effects of Mg doping on the remarkably enhanced electrochemical performance of Na3V2(PO4)3 cathode materials for sodium ion batteries, Journal of Materials Chemistry A 3(18) (2015) 9578-9586.
[18] H. Li, H. Tang, C. Ma, Y. Bai, J. Alvarado, B. Radhakrishnan, S.P. Ong, F. Wua, Y.S. Meng, C. Wu, Understanding the Electrochemical Mechanisms Induced by Gradient Mg2+ Distribution of Na-Rich Na3+xV2–xMgx(PO4)3/C for Sodium Ion Batteries, Chemistry of Materials 30(8) (2018) 2498-2505.
[19] Y. Chen, J. Cheng, Y. Wang, C. Wang, Z. He, D. Li, L. Guo, Insights into the elevated electrochemical performance and kinetic characteristics of magnesium-substituted Na3V2−xMgx(PO4)3/C with superior rate capability and long lifespan, Journal of Materials Science 55(27) (2020) 13141-13156.
